# Implementation of simple and effective fine droplet formation-based spray-assisted liquid phase microextraction for the simultaneous determination of twenty-nine endocrine disruptor compounds and pesticides in rock, soil, water, moss, and feces samples from antarctica using gas chromatography-mass spectrometry

**DOI:** 10.1007/s11356-023-31750-8

**Published:** 2024-01-12

**Authors:** Buse Tuğba Zaman, Gamze Dalgıç Bozyiğit, Meltem Şaylan, Elif Seda Koçoğlu, Bedrihan Kartoğlu, Efe Sinan Aydın, Ayça Girgin, Tülay Borahan, Sude Oflu, Yağmur Kılınç, Emine Gülhan Bakırdere, Sezgin Bakırdere

**Affiliations:** 1 Department of Chemistry, Faculty of Art and Science, Yildiz Technical University, 34220 İstanbul, Türkiye; 2 Department of Environmental Engineering, Faculty of Civil Engineering, Yildiz Technical University, 34220 İstanbul, Türkiye; 3 Department of Pharmacy, İstanbul Health and Technology University, Seyitnizam Street, No.: 85, İstanbul, Türkiye; 4 Yildiz Technical University Central Research Laboratory, 34220 İstanbul, Türkiye; 5 Department of Chemical Engineering, Faculty of Chemistry and Metallurgy, Yildiz Technical University, 34220 İstanbul, Türkiye; 6 Neutec Pharmaceuticals, Yildiz Technical University Technopark, 34220 Istanbul, Türkiye; 7 Department of Environmental Engineering, Institute of Science, Zonguldak Bülent Ecevit University, 67100 Zonguldak, Türkiye; 8 Department of Science Education, Faculty of Education, Yildiz Technical University, 34220 İstanbul, Türkiye; 9 Turkish Academy of Sciences (TÜBA), Vedat Dalokay Street, No. 112, Çankaya, 06670 Ankara, Türkiye

**Keywords:** Antarctic region, Endocrine-disrupting compounds, Gas chromatography, Moss, Seawater, Soil

## Abstract

This study established the simultaneous determination of the selected endocrine-disrupting compounds (EDCs) and pesticides in rock, soil, water, moss, and feces samples collected from the Antarctic region. The spray-assisted droplet formation-based liquid phase microextraction (SADF-LPME) coupled to GC–MS system was developed and validated for the screening and monitoring of 29 selected EDCs and pesticides. Binary solvent system, 1:1 (v/v) dichlormethane: 1,2-dichloroethane mixture was employed as an extraction solvent and sprayed onto sample or standard solutions using a straightforward and practical spray apparatus. The factors affecting the extraction process such as extraction solvent type and ratio, extraction solvent volume (spray repetition), vortexing period, and sample pH were properly optimized. Analytical figures of the merit of the method were recorded under the optimal extraction/chromatographic conditions. The LOD, LOQ, and enhancement factor were in the range of 1.0 to 6.6 ng/g, 3.2 to 22.1 ng/g, and 3.7 to 158.9, respectively. The method demonstrated a good linear working range for all the selected analytes with proper coefficients of determination. The usability and reliability of the microextraction strategy was confirmed using seawater, moss, and soil samples, and the %recoveries were within an acceptable range (> 70%) for all examined samples. The environmental samples collected from the Horseshoe and Faure Islands of the Antarctica region were analyzed to assess the potential pollution of EDCs and pesticides. This method has the potential to be employed for the analysis of EDCs in routine analytical laboratories and for controlling and screening the organic pollutant content of different environmental samples.

**Supplementary Information:**

The online version contains supplementary material available at 10.1007/s11356-023-31750-8.

## Introduction

One of the primary concerns in the twenty-first century is the occurrence and wide distribution of persistent organic pollutants (POPs) and emerging contaminants (ECs) in the environment (Ros et al. 2015). Among these pollutants, endocrine-disrupting compounds (EDCs) have raised concerns due to their negative impacts on public health and the environment (Ros et al. 2015) (Zhang et al. 2012). EDCs are exogenous compounds that get involved in the endocrine system with their ability to disrupt the metabolic pathway of hormones and antagonize or mimic natural hormones such as androgens and estrogens (Scognamiglio et al. 2016)(Petrović et al. 2001). Exposure to these diverse pollutants occur via different sources such as personal care products, food pollutants, waste effluents, therapeutic agents, environmental samples, and consumer products (Robitaille et al. 2022). According to the literature researches, exposure to endocrine-disrupting compounds causes lifelong health problems for humankind and living things. They cause obesity, abnormalities in growth, neurological problems, and cancers related to hormones and disturb the reproductive systems in humans. Damaged nervous, reproductive, and immune systems in wildlife have been reported related to the toxicity of EDCs (Vieira et al. 2021). Even if they are present at sub-μg/L levels, they can cause negative effects to aquatic life, environment, and human health (Nasir et al. 2022). Accurate monitoring of the low levels of these contaminants requires sensitive and accurate analytical methods.

Similarly, the issue of pollution caused by pesticide mixtures is gaining prominence on a global scale due to the potential for these mixtures to induce synergistic toxicity in non-target organisms during both acute and chronic exposures (Maloney et al. 2018). According to the United Nations Organization for Food and Agriculture (FAO) definition, pesticides are a substance or combination of substances designed for the purpose of cultivating, eradicating, preventing, or managing any form of pest, including vectors of human or animal diseases, undesired species of plants or animals that cause harm, or any other interference in the production, storage, transportation, processing, or marketing of food, agricultural products, wood and wood products, or animal feed. Additionally, these substances may be administered to animals for the purpose of controlling insects, arachnids, or other pests residing on or within their bodies (Akashe et al. 2018). The EU Pesticides Database contains a total of 1378 active ingredients, with 466 of these being approved for use within the European Union, while the remaining 858 have not yet received approval for usage in the EU (Kalyabina et al. 2021). Pesticides exhibit varying distribution and persistence patterns within the environment, despite their universal distribution through air, soil, and water pathways (Gaikwad and Mairal 2022). Organochlorine pesticides (OCPs), a class of pesticide derivatives extensively employed in agricultural practices during the 1950s (Thompson et al. 2017), have currently been classified as POPs and are prohibited in the majority of nations, in compliance with the regulations outlined in the Stockholm Convention (Fiedler et al. 2019). However, agricultural soils continue to exhibit high concentration levels of pesticides, as well as their transformation products and degradation products. Inevitably, the identification of low levels of pesticides is crucial in assessing potential hazards, particularly within the food chain and water resources.

Antarctica is considered an untouched region free from pollutants and unaffected by anthropogenic activities (Bargagli 2005). Contrary to what is considered, it is actually under the influence of anthropogenic activities from logistic operations, local activities, and scientific stations (de Lima Neto et al. 2017). Development of tourism and scientific stations impacted the naval coastal and local continental ecosystems via accidental oil spills, sewage, fuel combustion, and waste incineration (Martins et al. 2004) (Bargagli 2008). Reported data on the amounts of persistent pollutants, metals and pesticides in marine organisms, snow, lichens, air and mosses demonstrate that most of these pollutants are carried from the other mainland in the Southern Hemisphere (Bargagli 2008). Even when no application has ever been done on the mainland some pesticides were detected in marine biota and abiotic samples in Antarctic (Esteban et al. 2016). Some pesticides including the EDCs such as toxaphene, hexachlorobenzene, chlordecone, endrin, dichlorodiphenyltrichloroethane (DDT), chlordane, dieldrin, heptachlor, lindane, and pentachlorobenzene have been suggested to be under control in the 12th Stockholm Convention (Potapowicz et al. 2020). In spite of the establishment of limitations and bans, the presence of these pesticides in the aquatic environment and trophic networks of the local organisms in the Antarctic has been reported by many researchers (Chiuchiolo et al. 2004; Dickhut et al. 2005; Taniguchi et al. 2009; Potapowicz et al. 2020). It is therefore of great importance to investigate the geographical distribution of endocrine-disrupting compounds in the Antarctic region using sensitive and accurate analytical methods.

Various analytical methods are used to detect and quantify organic compounds in environmental samples with high accuracy and precision. Determining these contaminants at trace levels in environmental samples requires additional sample preparation techniques before their determination with sensitive instruments (Locatelli et al. 2016)(Huerta et al. 2015)(Mijangos et al. 2015). Microextraction methods have become very popular for the separation/preconcentration of organic and inorganic analytes at trace levels due to their great advantages such as simplicity, rapidity, and reduced organic solvent consumption (Zhao et al. 2020). Liquid phase microextraction (LPME) methods including single-drop microextraction (SDME), switchable solvent microextraction (SS-LPME), ionic liquid phase microextraction (IL-LPME), cloud point extraction (CPE), and dispersive liquid–liquid microextraction (DLLME) have been applied for the determination of EDCs in various sample matrices (Kandhro et al. 2012, 2014; Chormey et al. 2020).

Dispersive liquid–liquid microextraction method is fast, easy to apply, affordable, and ecologically friendly and can provide high enrichment over a variety of acceptor and donor phases (Rykowska et al. 2018). The traditional dispersive liquid–liquid microextraction method is based on the dispersion of the extraction solvent in the form of tiny droplets throughout the aqueous sample solution with a dispersion solvent (Regueiro et al. 2008). Nevertheless, using high volumes (mL level) of dispersive solvent results in a reduced extraction efficiency by enhancing the solubility of lipophilic analytes in the aqueous solution. Moreover, utilizing high volumes of dispersive solvents such as methanol is not environmentally friendly (Farajzadeh and Mogaddam 2012)(Jiang et al. 2014). Therefore, different methods have been developed to eliminate the need for a disperser solvent and assist the dispersion using surfactants (Tseng et al. 2014), vortex (Yiantzi et al. 2010), effervescent tablets (Borahan et al. 2021), automatic shaking (Chen et al. 2014), ultrasound (Regueiro et al. 2008), and spraying apparatus (Dikmen et al. 2020).

Spray-assisted droplet formation-based liquid phase microextraction (SADF-LPME) method is a dispersive-solvent-free method which is a modified version of DLLME method in accordance with green analytical chemistry. This method assists the dispersion by a simple apparatus which is used to spray the extraction solvent as fine droplets throughout the aqueous sample solution. Therefore, the amount of organic solvent used for dispersion in the traditional DLLME methods is reduced and the experimental procedure becomes more rapid (Dikmen et al. 2020).

This study presents a spray-assisted droplet formation-based liquid phase microextraction method for the separation/preconcentration of 29 trace endocrine disrupting compounds and pesticides before their determination by gas chromatography–mass spectrometry in environmental samples collected from the Faure and Horseshoe Islands during the 4th National Antarctic Science Expedition of Türkiye. The developed method is a dispersive-solvent-free method which eliminates the need for a disperser solvent. Instead of applying traditional solvent dispersers, a simple and easily affordable apparatus was used for the dispersion procedure which makes the method more environmentally friendly. Recovery experiments were applied to seawater, lake water, moss, feces, and rock-soil samples to validate the applicability and accuracy of the developed method.

## Material and methods

### Chemicals and reagents

All chemicals and reagents worked throughout the study were preferred of analytical quality purity. The reference standards of selected pesticides and hormones were purchased from Sigma Aldrich and Dr. Ehrenstorfer (Germany). In Supporting Information Table S1, the target EDCs are listed with their specific characteristics and main quantifier ions. Stock standard solution of each analyte was separately prepared using HPLC-grade acetonitrile (98–101%, Merck). The stock solutions of analytes were used to prepare mix stock solutions containing the selected EDCs at the desired concentrations. The mixed stock/sample solutions were gravimetrically and daily prepared throughout the study. All standard/sample preparations were kept at a − 22 °C freezer kept out of moisture and sunlight. Stock solutions were diluted to prepare working/calibration solutions for optimization steps with deionized water (conductivity, 18.2 MΩ cm^−1^) obtained from the ELGA Pureflex III (London, UK) ultrapure water treatment system. Dichloroethane, dichloromethane, and chloroform used as extraction solvent throughout the study were purchased from Merck, Germany. Disodium tetraborate decahydrate (Merck, Germany) was utilized to prepare borate buffer solution (pH 9.0). HCl solution (37%) and sodium hydroxide was purchased from the Merck, Germany. In the study, 99.995% pure helium (He) gas employed as a carrier gas in the GC–MS system was supplied from a gas supply company in İstanbul, Türkiye.

### Instrumental and chromatographic conditions

Chromatographic separations and mass selective detections for the selected EDCs were accomplished by an Agilent 6890 GC system coupled with an Agilent 5973 mass spectrometry detector. The target compounds were separated using HP-5MS (Agilent, USA) capillary column (30 m × 0.32 mm i.d., 0.25-μm film thickness). The standard/sample solutions were sent to the system using HP 6890 model autosampler and an SGE Analytical Science (Melbourne, Australia) microinjector. All injections were performed in the splitless mode with a standard/sample volume of 2.0 μL at a constant column flow rate of helium (0.8 mL/min). In the sample introduction port, a deactivated inlet liner was equipped. This liner has 4.0 mm and 6.47 mm inner and outer diameter, respectively. It has a single taper shape which has glass wool at the junction point with the analytical column. It has 900 µL total volume with 78.5-mm length. The purge flow was set to 60 mL/min with 1.0-min purge time. The ionization energy of the MS detector, MS quadrupole temperature, and ion source temperature was 70 eV, 150 °C, and 230 °C, respectively. The injector temperature and mass transfer line temperatures were set as 250 °C and 290 °C, respectively. The oven temperature program was as follows: initial oven temperature, 100 °C, 15 °C /min rate to 200 °C (held for 1.0 min), 5.0 °C/min rate to 280 °C (held for 1.0 min), and 10 °C/min rate to 300 °C within 2.0 min. The selected EDCs were separated and eluted at different retention times with the set operating chromatographic conditions, and the total run time was 27 min. Fig. S1 displays the EDCs’ total ion chromatogram (TIC) with analyte number, which includes the analyte peaks. The analyte numbers, retention times, molecular weights, and the prominent ion fragment (m/z) of each analyte are also listed in Supporting Information Table S1. SCAN mode was employed to acquire chromatographic data for all analyses in the m/z range of 40 to 600. Data acquisition, instrument control, and processing were carried out with ChemStation® software.

OHAUS Pioneer PA214C model analytical balance was utilized in the preparation of calibration and working samples. ISOLAB brand vortex (Dramstadt, Germany) and Kermanlar brand mechanical shaker (İstanbul, Türkiye) were employed for mixing processes in the extraction procedure. BioBase brand ultrasonic bath (Shandong, China) was also used in the real sample preparation procedure. The BIOBASE BKCTL5II centrifuge (Shandong, China) was utilized in facilitating the phase separation.

### Design of the spray apparatus

**Fig. 1:**
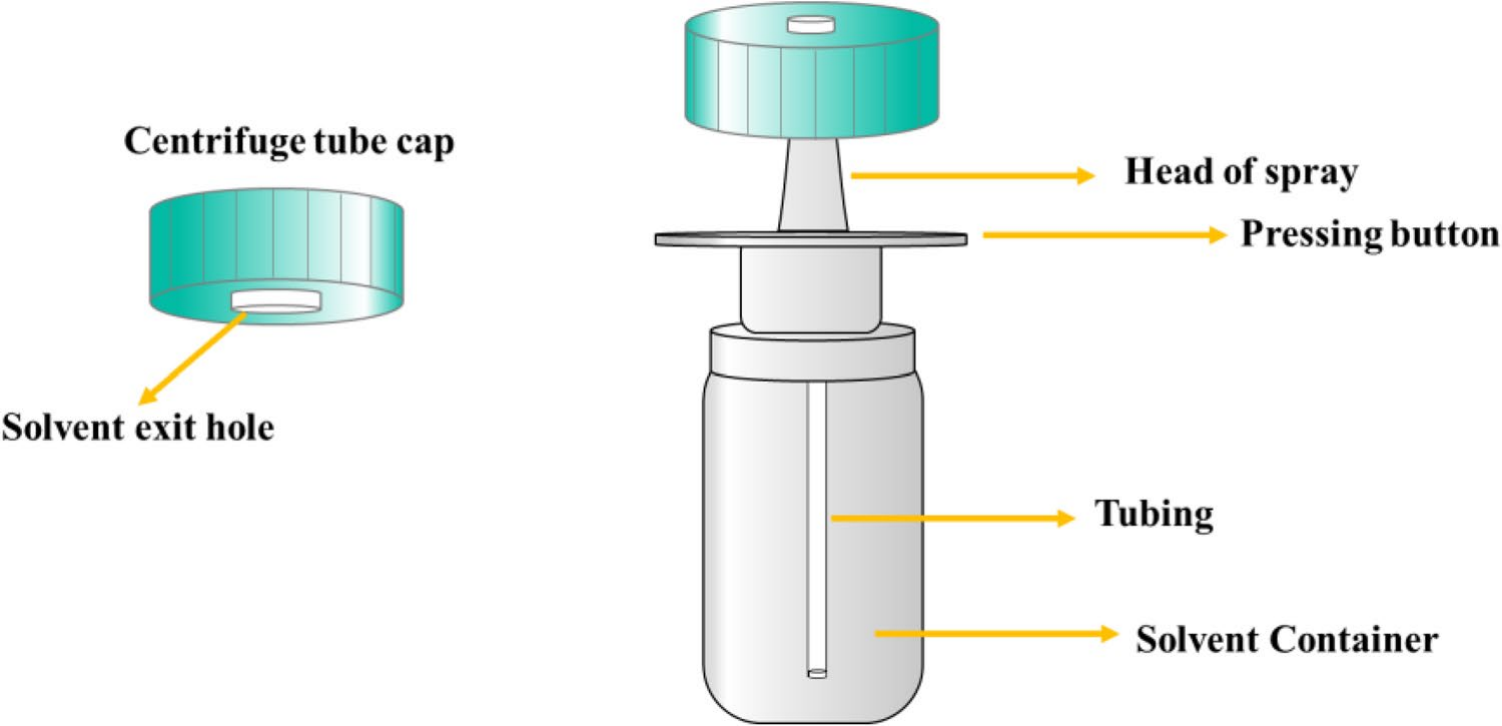
The design of the modified spray system for the formation of droplets

The extraction solvent mixture containing a mixture of DCM and DCE was dispersed uniformly and efficiently into the aqueous solution using a simple spray system. The extraction solvent was successfully dispersed into the samples without using the dispersion solvent with this presented spray system. In this regard, the experimental steps and solvent usage were minimized compared to the classical DLLME method. The spray apparatus was provided by a nearby pharmacy and was easily accessible. The spray system consisted of a centrifuge tube cap, a spray head, a transfer tube for transferring the extraction solvent to the spray head, and an extraction solvent container. The design of the spray apparatus is explained in detail in previous studies (Erulaş et al. 2020). The extraction solvent container had a 20-mL internal volume. The relative errors caused by the injection and experimental procedures were minimized by using the spray system. The repeatability of each spray cycle was controlled using an analytical balance. The DCM-DCE (1:1, v/v) mixture was sprayed into clean centrifuge tubes with 15 replicate measurements. As a result, the average amount of extraction solvent mixture consumed in one spray cycle was recorded 0.1326 g with a 0.0045 g standard deviation. The spray system is represented in Fig. 1.

### Spray-assisted droplet formation-based liquid phase microextraction procedure

**Fig. 2:**
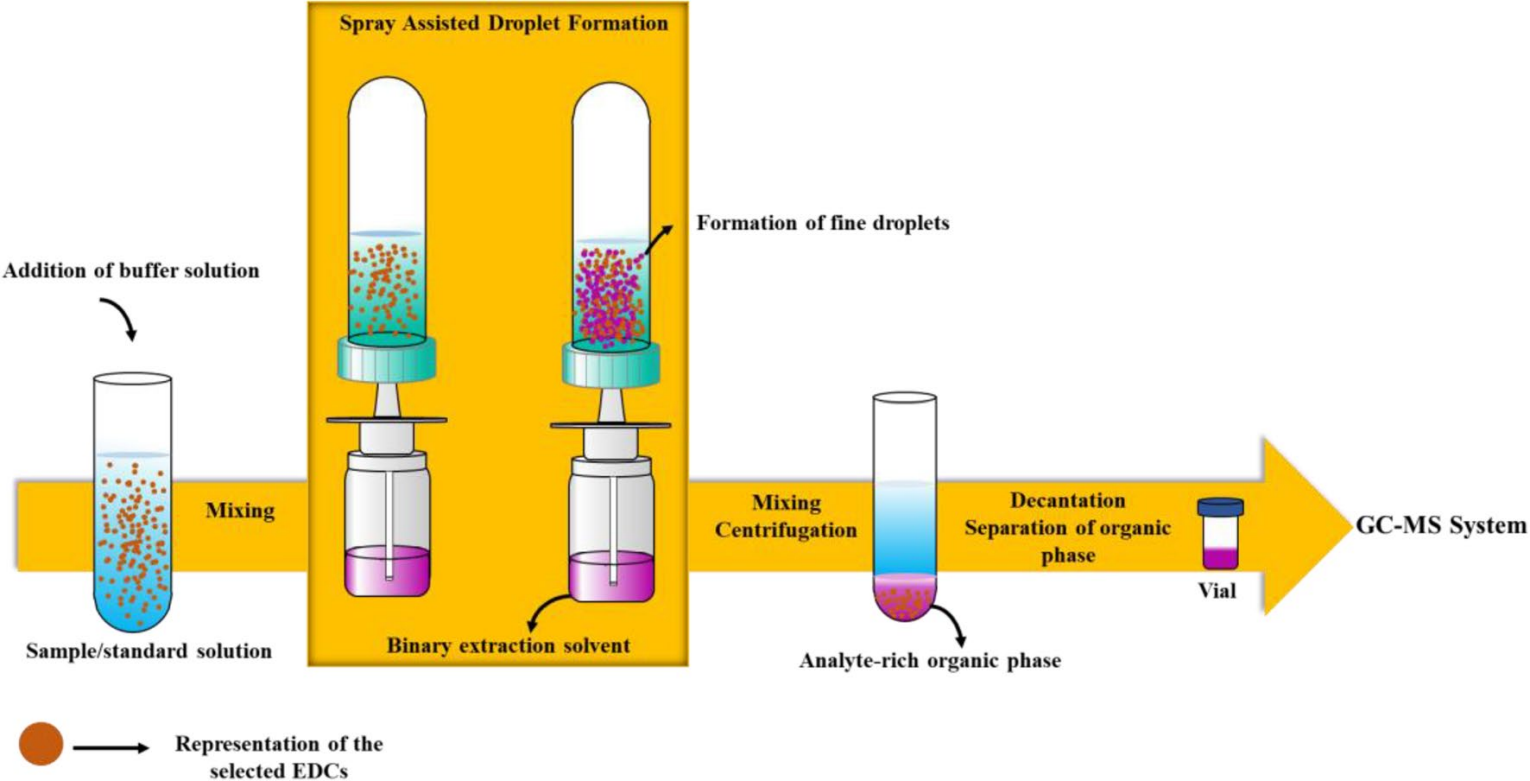
Illustration of SADF-LPME strategy for the examined EDCs and pesticides

In the purposed method, the proper amount of the aqueous solution spiked with target analytes was transferred into a clean 15-mL test tube with conical bottom, and 0.50 mL of 50 mM borate-HCl buffer solution (pH 9.0) was added into it. Afterward, the samples were mixed with a vortex for 5.0 s to ensure effective distribution of the buffer solution into the working solutions. Then, the binary solvent containing 1,2-dichloroethane and dichloromethane at a ratio of 1:1 (v/v) was dispersed in two spray cycles into the working solutions using the customized spraying apparatus described in the relevant section above. The solution was mixed with the help of a vortex for 15 s to accelerate the interaction of the extraction solvent with the analytes. The resulting cloudy solution was centrifugated at 3460 g for 2.0 min. Subsequently, the analyte-rich organic phase was collected at the conical bottom of the test tube. Then, the clear aqueous phase remaining on top was carefully removed with a pipette. As a result, the organic phase was analyzed by a GC–MS instrument. The samples was stored in a freezer at − 16 °C before the sampling into the GC–MS system. At least two replicate readings were used in each experiment. The schematic illustration of SADF-LPME method is given in Fig. 2.

### Sample collection and preparation

The 200 samples (92 rock-soil, 43 moss and seaweed, 42 seawater, 12 lake water, and 11 penguin and seal feces) were collected on the Horseshoe Island and Faure Island of Antarctica during the 4th National Antarctic Science Expedition of Türkiye in February–March 2020. Samples were collected and stored in clean polyethylene bottles. The seaweeds, feces, and moss samples were kept in a deep freezer at − 16 °C. The other collected samples were stored in a refrigerator at 4.0 °C. Details about the collection and preparation were described in the previously published study by Tekin et al. (Tekin et al. 2022). Accordingly, extraction procedure was employed for solid/rock, seaweed, and fece samples, and the sample extracts in acetonitrile were obtained to use in the real sample application processes. The preparation procedures for each sample that were used in the recovery experiments are described briefly below.

#### Seawater samples

Two different seawater samples were collected from around Horseshoe Island of the Antarctic Continent. Seawater samples, coded HS-D-7 and HS-D-26, were randomly selected and used in recovery studies. The seawater samples were stored at + 4.0 ± 0.8 °C in completely closed, clean polypropylene containers away from sunlight. A mass-based preparation was implemented to prepare each sample. Aqueous samples containing mixed standard solutions at concentrations ranging from 20 to 500 ng/g were prepared by sampling 0.80 g of seawater samples and diluting them at 8.0 g with deionized water. The quantity of acetonitrile in all samples was maintained at 1.0% (w/w%).

#### Soil samples

Soil samples A and B were collected from different locations in İstanbul, Türkiye. These collected samples were employed for the preparation of blank and spiked samples. Firstly, 20–25 g samples were weighted and dried in an oven at 55–60 °C for two days to remove water and moisture. The dried soil was ground using a blender, then passed through a sieve with a pore diameter of 100 µm. In a clean polypropylene test tube, 5.0 g of the dried soil samples were then measured out, and 25 g of acetonitrile was added to the soil samples. The soil extracts were vortexed for one minute and then mixed in a mechanical shaker for 45 min. At the end of the mixing process, the sample solution was sonicated for 10 min using an ultrasonic bath (at a frequency of 45 mHz) for the extraction. The extracts were then centrifuged at 3000 rpm for 2.0 min, and solid particles were collected at the bottom of the centrifuge tube. The resulting extracts were finally filtered using filter paper with a 110-mm pore diameter. Aqueous soil extracts were prepared gravimetrically, and the amount of organic solvent (acetonitrile) was kept at 1.0% (w/w%) in the aqueous sample solutions used in the microextraction method. For this purpose, a standard addition process was applied after the soil sample extracts were weighed at 0.80 g, and the solvent was completely removed by volatilization at ambient temperature in a fume hood. In aqueous samples, acetonitrile was added to assure that the amount of organic solvent was 1.0% and spiked at different concentrations (20 to 500 ng/g), and then, each sample was diluted with 8.0 g of deionized water. Before the standard additions, the resulting sample extracts were kept at closed polypropylene test tubes in a freezer at − 20 °C.

#### Seaweed samples

The two representative moss samples were used in recovery experiments due to the limited number of seaweed samples collected from around Horseshoe and Faure Islands. For this purpose, two representative moss samples (A and B samples) were taken from different regions in İstanbul, Türkiye, for method validation. The collected moss samples were repeatedly washed with tap water to prevent possible contamination or impurites. These processes were then repeated with deionized water. The washed moss samples were dried in an oven at 55–60 °C for two days. Dried moss samples were pulverized in a mortar. Then, 40 g of acetonitrile was added to 8.0 g of moss sample, and aqueous moss extract were prepared in the same process as the extraction applied to soil samples. Moss samples were stored in sealed polypropylene test tubes in a freezer at − 20 °C.

### Method validation

The analytical performance of the method was examined in accordance with the validation factors, including limit of detection (LOD), limit of quantification (LOQ), linearity, precision, and accuracy. The peak areas were calculated with the MSD ChemStation® software program in the chromatograms obtained by using the m/z ratio ion with the highest relative abundance of each analyte. The linearity for all the selected EDCs in standard solution or matrix was obtained by plotting the peak area against the corresponding standards at different concentrations. A calibration curves/plots were achieved by extracting a series of mixed standard EDC and pesticide solutions prepared in acetonitrile. LOD and LOQ values were calculated using the following Eqs. (1) and (2):

1 $${\text{LOD}}={3}^{*}{\text{SD}}/m$$

2 $${\text{LOQ}}={10}^{*}{\text{SD}}/m$$

where SD is the standard deviation in the recorded (signal-to-noise ratio ≥ 3) lowest spike level of replicate measurements, and *m* is the slope obtained from the calibration plot. In order to determine the precision of the developed method, all samples were prepared at least 2 replicates and 4 measurements for each sample set were performed. The standard deviation of this sample sets were divided by the average value to determine the %RSD value. In order to determine the effectiveness of the developed method, the improvement in detection power was calculated. This calculation was based on the ratio of the limit of detection obtained by direct GC–MS measurements of the analyte (LOD_GC-MS_) to the limit of detection obtained by analyzing the samples prepared after extraction in the GC–MS system. The matrix matched standards of soil, seawater, and moss samples were employed to calculate recoveries, and the recovery experiments were also performed on control (blank) samples of seawater, lake water, soil, rock, feces and seaweeds samples collected from Horseshoe and Faure Islands.

## Results and discussion

### Selection of the proper extraction method

The effect of different sample preparation methods on extraction performance was determined for the simultaneous preconcentration/separation of the investigated analytes from environmental samples. Accordingly, the extraction efficiency and selectivity were tested by three different microextraction techniques including switchable solvent-based liquid phase microextraction (SS-LPME), dispersive liquid–liquid microextraction (DLLME), and spray-assisted droplet formation-based liquid phase microextraction method (SADF-LPME). The average enhancement factors in analyte signals determined by SADF-LPME, DLLME, and SS-LPME techniques were found to be 50.3, 40.81, and 41.25 folds, respectively. The SADF-LPME method exhibited a good extraction capability and chromatographic peak shape under the defined chromatographic conditions, making it more effective and practical than other applied sample preparation methods. As a result of reducing the number of extraction steps, it may cause the errors arising from the sample preparation decreased with SADF-LPME method. Hence, this method was selected as an ideal sample preparation method to develop an accurate and sensitive analytical strategy and to introduce a more practicable, affordable, and eco-friendly method for the literature.

### Optimization of the SADF-LPME strategy for the selected EDCs

The experimental parameters affecting the extraction performance of the selected 29 EDCs and pesticides including the type and ratio of extraction solvent, the pH of the sample solution, and spray repetition were determined by one variable at a time approach. The chromatographic signals were utilized to examine the effect of the variables on the extraction efficiency by the SADF-LPME in GC–MS system. In the optimization steps, the best extraction condition was determined by using 5.0 mL of 500 ng/g test solutions, and other studies were accomplished with 8.0 mL of fixed sample solution. The average peak areas and standard deviations of replicate readings were computed and employed to define the best extraction conditions.

#### Effect of the sample pH

The mass transfer of analytes from aqueous solution to organic solvent is affected by pH, which has an impact on the extraction performance. In this step, the effect of pH was checked using a 1.0-mL buffer solution at pH 3.0, 5.0, 7.0, and 9.0, as well as natural pH condition with no external intervention (Fig.S2). According to the findings from the buffered solutions, it is clear that there is no big cnages in the results obtained at different pH values. It is seen that the most of the analytes were successfully extracted with a borate-HCl buffer solution (pH 9.0). However, some analytes with high signals in the acidic region (pH 3.0) had 1.0–1.2 times lower signals than in the basic conditions (pH 9.0). Ignoring this difference, the pH 9.0 value, which offers high extraction outputs of most analytes, was determined as the optimal extraction pH.

Additionally, 0.25, 0.50, 1.0, and 2.0 mL of a 50 mM borate-HCl buffer solution were studied to assess the influence of buffer amount. The signals were significantly decreased with increasing volumes due to the dilution of samples. Results showed that 0.25 mL of borate-HCl buffer was sufficient for the extraction of 17 analytes with the highest peak areas (Fig. S3). However, the ideal amount of buffer solution was determined to be 0.50 mL to provide better buffering capacity for the samples analyzed.

#### Effect of extraction solvent type and ratio

Ideally, the selected extraction solvent should have specific properties such as the high capability for the target analytes, higher or lower density than water, immiscible in water, good chromatographic behavior, and ease of dispersion into the water during the dispersing step (Jouyban et al. 2020). Accordingly, the extraction performance of the method was examined with water-immiscible organic solvents including chloroform (CHL), dichloromethane (DCM), and 1,2-dichloroethane (DCE), as well as binary and ternary combinations of these extraction solvents (CHL-DCM, CHL-DCE, DCM-DCE, and CHL-DCM-DCE).

The binary (1:1, v/v) and ternary (1:1:1, v/v) combinations of solvents were prepared at fixed ratios in this optimization, and all mixtures were vortexed at 5.0 s. Results showed that CHL-DCM achieved the highest extraction outputs for 18 analytes and DCE-DCM for 12 analytes. However, the relative standard deviation values obtained for the analytes with the highest peak area in the CHL-DCM mixture revealed low repeatability. Contrarily, the %RSD values for the DCE-DCM mixture were below 10% for each analyte. Additionally, the average peak area values obtained from the mixture of DCE-DCM for the analytes with the highest extraction output in the mixture of CHL-DCM solvent revealed also an insignificant difference, with results of 1.0–1.3 times higher. Hence, DCE-DCM mixture was chosen as optimal extraction solvent for better extraction capability in subsequent steps.

**Fig. 3:**
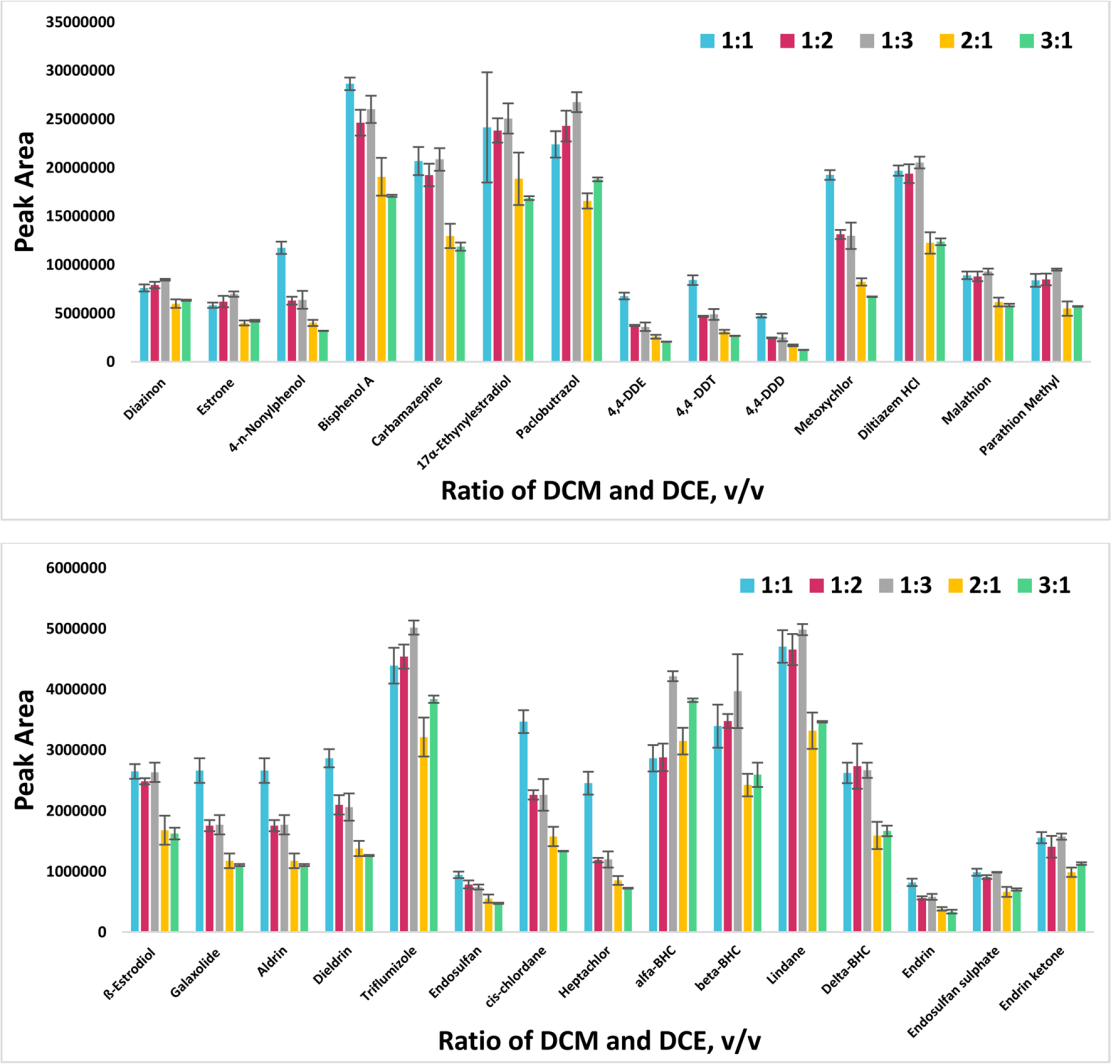
Effect of the extraction solvent ratio for the determination of the selected EDCs (Conditions: 5.0 mL of sample solution, 0.50 mL borate-HCl buffer solution at pH 9.0, 2 spray repetition of DCE-DCM mixture, and 15 s vortex, *n* = 4)

The ratio of DCE-DCM mixture significantly affected the extraction efficiency. In this regard, the experiments were carried out with two spray repetitions, and different ratio of DCE-DCM mixture including 1:1, 1:2, 2:1, 1:3, and 3:1 (v/v). The corresponding results are represented in Fig. 3. According to the experimental data, repeatability noticeably decreased at high DCM ratios. It can be related to the high solubility of the solvent in aqueous solution (1.6%) (Chormey et al. 2022). This causes the difficulty of sufficient amount analyte-rich phase separation for quantitative analysis. The findings indicated that 1:1 (v/v) ratio of the DCE-DCM was the most efficient extraction solvent for target analytes in the proposed method and, therefore, selected for the subsequent experiments.

#### Optimization of spray number to determine extraction solvent volume

The extraction solvent volume is directly related to the extraction outputs. Complete separation of the organic compounds from environmental samples is provided by sufficient extraction solvent. The smaller volume of DCE-DCM solution is linked to high extraction efficiency and the least hazard to environmental. However, it is challenging to completely extract the analytes and collect the extraction solvent when the volume of extraction solvent is too small. In this study, the simple spray apparatus introduced in “Design of the spray apparatus” was employed to ensure the extraction solvent to be dispersed into the aqueous phase as fine droplets to form a cloudy solution. Each spray repetition was proportional to extraction solvent volume. Hence, different spray repetition including one, two and three squeezed number, were studied to evaluate ideal extraction solvent volüme (Fig. S4). The results demonstrated that the signals of EDCs were gradually declined with higher spray number due to the dilution of analyte at the final volume. Although the highest outputs were observed with one spray repetition, the repeatability was decreased, and phase separation was difficult due to collection of organic phases. Considering practicability and analytical signals, two spray cycle were chosen for further studies.

### Analytical performance value of the presented method

The linearity, sensitivity, accuracy, LOD, and LOQ values were studied to check the performance of the presented extraction strategy. The GC–MS calibration plots demonstrated a high coefficient of determination (*R*^2^) in the range of 0.9983–1.000, indicating that high linearity was attained in the determination of analytes under ideal chromatographic conditions. The target analytes' LOD and LOQ values for the GC–MS system were established to be between 3.6 and 419.7 ng/g and 12.0 and 1399 ng/g, respectively. All 29 analytes recorded relative standard deviations (%RSD) that were lower than 18%, indicating the sufficient precision for replicate instrumental readings. Accordingly, the extracted EDCs were analyzed by GC–MS. The analytical performance of the GC–MS system for simultaneous detection of analytes is detailed in Supporting Information Table S2.

The calibration plots for all the EDCs were formed using different standards in the range of approximately 0.05–100 mg/kg that were extracted under optimum extraction conditions (Table S3). The method exhibited a good linearity with *R*^2^ ranging between 0.9830 and 1.000. In the combined method of SADF-LPME-GC–MS, LOD, and LOQ values were found in the range of 1.0–6.6 ng/g and 3.2–22.1 ng/g, respectively. The enhancement of detection power (EDP) was calculated by comparing the LODs of each investigated system. For this reason, the LOD values of proposed method was compared with LOD values of GC–MS system. Accordingly, the EDP values were noted for the target analytes in the range of 3.7 to 158.9. The analytical figures of merit of the SADF-LPME-GC–MS are presented in Table 1.

**Table 1:** Analytical figures of merit of the SADF-LPME-GC–MS combined method for EDCs

Analyte	LOD (ng/g)	LOQ (ng/g)	Linear range (ng/g)	*R*^2^	%RSD	EDP
Alpha-BHC	6.6	21.9	13.8–205.0	0.9950	7.2	3.7
Beta-BHC	2.4	7.9	6.4–205.0	0.9991	3.8	10.1
Lindane (gama-BHC)	2.6	8.7	8.1–205.0	0.9958	12.5	5.4
Diazinon	1.4	4.5	4.8–89.7	0.9987	8.0	19.1
Delta-BHC	5.4	17.9	13.8–205.0	0.9942	6.4	16.8
1,3,4,6,7,8-Hexahydro-4,6,6,7,8,8-hekzame (galaxolide)	1.3	4.3	3.8–154.4	0.9961	10.9	56.0
4-n-Nonylphenol	1.0	3.3	4.7–194.7	0.9940	16.4	31.8
Parathion methyl	1.2	4.1	4.8–90.1	0.9979	8.9	62.3
Heptachlor	2.3	7.5	8.0–202.6	0.9937	19.4	10.6
Malathion	1.4	4.8	4.8–197.1	0.9991	8.3	29.0
Aldrin	3.8	12.6	13.2–201.3	0.9987	8.1	9.8
Triflumizole	2.7	9.1	12.5–195.9	0.9976	14.1	14.3
Paclobutrazol	1.6	5.3	4.9–200.4	0.9981	6.8	27.9
cis-Chlordane	2.7	9.1	13.4–202.5	0.9996	6.1	15.0
Bisphenol A	1.4	4.7	4.8–499.3	0.9992	5.5	43.3
4,4-DDE	2.4	8.1	14.6–205.0	0.9830	4.5	7.8
Dieldrin	3.0	10.1	13.2–201.7	0.9955	5.3	9.2
Endrin	3.2	10.6	13.8–205.0	0.9922	5.5	16.7
Endosulfan	2.2	7.2	13.2–201.4	0.9972	3.1	57.2
4,4-DDD	1.5	5.0	13.8–205.0	0.9897	11.8	6.0
Endosulfan sulfate	1.4	4.5	4.8–89.7	0.9987	8.0	31.6
4,4-DDT	1.7	5.7	8.1–205.0	0.9931	17.1	53.6
Carbamazepine	1.3	4.2	4.9–504.0	0.9990	5.2	158.9
Endrin ketone	1.3	4.2	6.4–205.0	0.9990	6.3	49.3
Methoxychlor	1.1	3.5	13.8–205.0	0.9875	16.2	3.8
Estrone	3.5	11.8	12.6–198.6	1.00	8.5	80.6
ß-Estradiol	6.6	22.1	24.1–505.9	0.9986	9.4	63.5
17α-Ethinylestradiol	1.1	3.8	4.8–197.2	0.9990	5.3	50.0
Diltiazem hydrochloride	1.0	3.2	4.8–196.5	0.9949	10.0	24.7

### Recovery studies

A validation process was performed by establishing %recoveries for 29 EDCs and pesticides in different presentative seawater, soil, and moss matrices. For this purpose, the intended method for the selected experimental samples was applied and analyzed in the GC–MS system. To evaluate the practicability of the method, real sample solutions containing different concentrations of target analytes with spiking procedures were prepared based on the linear working range of the SADF-LPME-GC–MS method. The peak areas of the analytes spiked in the real sample and deionized water were compared, and the influence of the matrix interferences was shown to have a decreasing/increasing effect on the analyte signals. Accordingly, there are significant differences to be noticed between the signal from the real sample solution with the same concentration of analyte spiked and the signal from the aqueous standard solution. The matrix matching approach was thus selected to be employed in recovery studies for the elimination of possible matrix interferences from the sample matrices. Each selected samples were spiked at different concentrations to obtain calibration plots and the details was mentioned in “Sample collection and preparation.” All samples were studied as four replicates throughout the recovery experiments.

The equation derived from the spiked HS-D-7, soil A, and moss A samples’ calibration plots were used to calculate the %recoveries of the spiked HS-D-26, soil B, and moss B samples, respectively, whereas the equation derived from the spiked HS-D-26, soil B, and moss B samples calibration plot was used to calculate the %recoveries of the spiked HS-D-7, soil A, and moss A samples, respectively. The analytes determined in the study and the % recovery values of different concentrations obtained in HS-D-26 an HS-D-7 coded seawater samples are summarized in Table 2 and Supporting information Table S4, respectively. The recovery values of seawater samples were determined to be between 71 and 128%. The results demonstrated that the analysis of seawater samples could be performed with the described method. The low standard deviation values and great repeatability of the method are confirmed by the % recoveries. However, it was not possible to achieve recovery values for the endrin ketone and parathion methyl that were within the acceptable limits (70%), and therefore an exact matrix matching strategy was employed for both analytes. The recovery results obtained for soils A and B are presented in Tables S5 and S6. Herein, the %recovery values obtained for Soil A and B samples were quite similar to each other and were recorded as 73.7–130.3% and 74.1–130.8%, respectively. Additionally, exact matrix matching strategy was employed for parathion methyl. It is clearly seen that the method can be applied to determine and control the selected EDCs in soil samples.

**Table 2:** Percent recovery results using matrix matching approach on seawater sample coded HS-D-26 (*n* = 4)

Analyte	Spiked concentration (ng/g)	%Recovery ± SD
Alpha-BHC	100.7	95.8 ± 6.7
	170.6	106.5 ± 3.4
Beta-BHC	100.7	91.0 ± 5.4
	170.6	105.8 ± 4.5
Lindane (gama-BHC)	51.1	80.3 ± 1.2
	100.7	103.5 ± 3.8
	170.6	109.7 ± 1.4
Diazinon	97.7	94.9 ± 8.0
	196.7	108.3 ± 2.2
	472.5	106.1 ± 4.5
Delta-BHC	51.1	92.9 ± 6.9
	100.7	103.9 ± 2.5
	170.6	116.7 ± 2.8
1,3,4,6,7,8-Hekzahydro-4,6,6,7,8,8-hekzame (Galaxolide)	76.8	106.9 ± 2.0
	154.6	110.3 ± 2.6
	371.3	97.6 ± 3.5
4-n-Nonylphenol	96.8	95.8 ± 10.8
	194.9	113.8 ± 1.4
	468.1	96.3 ± 4.8
Parathion methyl *	49.1	95.4 ± 6.5
	98.2	101.0 ± 4.0
	197.8	106.0 ± 4.2
Heptachlor	100.1	108.0 ± 8.4
	185.5	117.7 ± 0.4
	443.1	77.6 ± 1.4
Malathion	49	105.6 ± 5.8
	98	114.1 ± 3.5
	197.4	112.7 ± 1.9
	474	99.1 ± 1.4
Aldrin	50.1	71.2 ± 3.7
	99.4	126.0 ± 1.3
	439.8	76.8 ± 1.5
Triflumizole	48.7	98.0 ± 6.2
	97.4	118.1 ± 8.2
	196.1	114.7 ± 1.9
	405	126.6 ± 13.1
Paclobutrazol	99.6	112.8 ± 1.4
	200	116.5 ± 5.4
	481.8	119.6 ± 5.3
cis-Chlordane	99.8	79.1 ± 4.3
	179.6	109.5 ± 0.8
	428	75.6 ± 2.6
Bisphenol A	49.1	114.7 ± 8.0
	98.2	110.9 ± 5.4
	197.7	98.5 ± 5.9
	478.8	100.1 ± 3.3
4,4-DDE	100.7	98.2 ± 3.2
	170.6	106.4 ± 2.6
Dieldrin	50.2	93.2 ± 1.2
	99.6	101.8 ± 2.8
	184.6	109.6 ± 2.0
	440.8	82.9 ± 1.0
Endrin	98.2	78.9 ± 8.6
	197.7	94.1 ± 1.0
Endosulfan	50.1	92.9 ± 2.1
	99.5	123.2 ± 6.0
	184.3	121.4 ± 2.1
	440.1	108.2 ± 1.4
4,4-DDD	100.7	96.0 ± 8.5
	170.6	111.5 ± 0.8
Endosulfan sulphate	51.1	97.7 ± 4.4
	100.7	103.7 ± 6.0
	170.6	113.3 ± 3.0
4,4-DDT	100.7	94.6 ± 6.7
	170.6	112.5 ± 1.6
Carbamazepine	49.6	112.1 ± 6.3
	99.1	113.1 ± 2.1
	199.6	109.0 ± 1.1
	479.4	105.6 ± 3.8
Endrin ketone*	51.1	89.1 ± 5.8
	100.7	95.0 ± 5.4
Metoxychlor	49.1	82.2 ± 9.0
	98.2	116.8 ± 8.3
	197.7	116.0 ± 2.4
Estrone	49.8	79.7 ± 2.7
	99.5	99.6 ± 6.8
	200.4	110.7 ± 5.3
	481.2	104.0 ± 2.9
ß-Estradiol	49.4	80.9 ± 3.1
	98.7	99.8 ± 6.8
	198.8	110.9 ± 5.2
	477.4	103.7 ± 2.2
17α-Ethinylestradiol	49	74.0 ± 4.5
	98	100.4 ± 5.8
	197.4	120.7 ± 2.7
	474	115.8 ± 4.7
Diltiazem HCl	49.1	84.9 ± 8.8
	98.2	95.8 ± 7.7
	197.7	114.5 ± 3.3
	474.8	110.3 ± 3.4

^*^Exact matrix matching was employed

The recovery values obtained for moss A and B, respectively, are listed in Tables S7 and S8. Herein, satisfactory recovery values were attained, like other sample matrices. The effect of matrix interferences on the analyte signals was determined for 4-n-nonylphenol, parathion methyl, heptachlor, 4,4-DDE, dieldrin, and endrin ketone. It was seen that the precise and accurate determination of these analytes was made compatible by using the exact matrix matching method. The recorded percent recovery values for moss A and B samples were found to be 77.6–126.9% and 75.9–132.4%, respectively.

### Application of the SADF-LPME-GC–MS method to environmental samples collected from Horseshoe and Faure Islands

The SADF-LPME-GC–MS method was employed to analyze 200 samples obtained from Antarctic Horseshoe and Faure Islands, and it was used to perform the qualitative and quantitative determinations of the selected 29 EDCs and pesticides. Aqueous standard solutions for samples from the Antarctic region were prepared according to the methods mentioned in “Sample collection and preparation.” All samples were extracted under optimal microextraction conditions, and then, analytes were all studied under appropriate chromatographic conditions. Analyte-specific signals for EDCs and pesticides were not detected in the examined samples according to the method detection limits. Samples were reanalyzed by adding a high concentration of a mixed standard solution of EDCs (5.0 mg/kg) to the real samples prepared for the examination of the matrix effect. The signals of a seaweed sample and the spike with a 5000 ng/g standard addition in the same seaweed sample are given in Fig. S5. The outputs proved that the analytes were determined with high accuracy and precision without notable matrix effect. All these results confirm that the purposed extraction strategy can be effectively used for the screening and monitoring of possible EDCs in Antarctic region.

### Comparison with the other methods

The quantitative characteristics and extraction efficiency of the method were compared with other reported extraction techniques for the selected pesticides and endocrine disrupting compounds in different sample matrices. In 2021, a dispersive liquid–liquid microextraction method coupled with GC–MS system were presented for the determination of eight potential endocrine disruptor pesticides (α-endosulfan, β-endosulfan, aldrin, diazinon, fenitrothion, lindane, malathion, and methoxychlor) in bovine milk samples. The LOD and recoveries values ranged from 0.90 to 5.00 ng/mL, and 86.15–112.45%, respectively (Sharma et al. 2021). The simultaneous extraction/determination of 17α-estradiol, 17α-ethinylestradiol, estrone, progesterone, testosterone, and phenolic compounds (4-nonylphenol and bisphenol A) in wastewater samples were reported by Ben Sghaier et al. (Ben Sghaier et al. 2017). The solid phase extraction method was applied, and the method LODs were in the range of 0.33–3.33 ng/L (Ben Sghaier et al. 2017). According to another study, the simultaneous determination of 79 pesticide residues (alpha-BHC, beta-BHC, diazinon, parathion-methyl, aldrin, etc.) in pigeonpea by LC–MS/MS and GC–MS/MS systems. The total analysis period was recoded at 40 min in the GC–MS/MS system. The reported method’s LOD and LOQ were 0.53–3.97 µg/kg and 1.60–10.05 µg/kg, respectively (Harischandra et al. 2021). In 2021, a dispersive micro-solid phase extraction method was introduced for the simultaneous extraction of 39 multiclass pesticides in environmental water including aldrin, 4,4-DDD, 4,4-DDE, and 4,4-DDT (Nascimento et al. 2021). The method ensured low LOD values, ranging from 0.51 to 22.4 ng/L, and an enrichment factor ranging between 72.5 and 200 (Nascimento et al. 2021). In comparison to the reported methods, this study indicated high repeatability, linearity, and rapid and easy extraction steps for the selected analytes. It can be concluded that the proposed extraction method combined with a GC–MS system could be applied for the selective and precise determination and monitoring of endocrine-disrupting chemicals including pesticides and hormones in environmental samples. The developed method can be combined with more sensitive instruments such as GC–MS/MS to lower the detection limits.

## Conclusion remarks

To the best of our knowledge, the combination of SADF-LPME-GC–MS methodology allowed, for the first time, the accurate and precise simultaneous determination of 29 EDCs and pesticides in seawater, seaweeds, and rock-soil samples collected from the Antarctic region. The chromatographic and extraction conditions were systematically optimized, and the SADF-LMPE method was employed to decrease extraction and dispersive solvent usage in accordance with the principles of *green chemistry*. The LOD and LOQ values for all analytes were recorded between 1.0 and 6.6 ng/g and 3.2 and 22.1 ng/g, respectively. The high enhancement factor was ensured by the spray-assisted extraction method at 3.7 to 158.9 folds. The method was validated using seawater, moss, soil samples, and environmental matrices collected from the Horseshoe and Faure Islands in the Antarctic region. The recoveries were found to be within an acceptable range for all studied samples. As a result, extraction capability and applicability have been confirmed by recoveries, ensuring satisfactory outputs for all examined EDCs. The GC–MS-based extraction method is applicable to detect and screen the selected EDCs at low concentration and can be used for continuous monitoring of pesticides or hormones in different areas and environmental matrices, especially in the Antarctic region.

### Supplementary Information

Below is the link to the electronic supplementary material.

Supplementary file1 (DOCX 374 KB)

## Data Availability

Data will be available upon reasonable request.
