# Supplementary material for: Implementation of simple and effective fine droplet formation-based spray-assisted liquid phase microextraction for the simultaneous determination of twenty-nine endocrine disruptor compounds and pesticides in rock, soil, water, moss, and feces samples from antarctica using gas chromatography-mass spectrometry
Source: Environ Sci Pollut Res Int. 2024 Jan 12;31(7):10920–33. doi: 10.1007/s11356-023-31750-8 (PMC10850215; doi:10.1007/s11356-023-31750-8)
Supplement: Supplementary file 1 — Supplementary file1 (DOCX 374 KB) [file 11356_2023_31750_MOESM1_ESM.docx]

**Implementation of Simple and Effective Fine Droplet Formation Based Spray Assisted Liquid Phase Microextraction for Simultaneous Determination of Twenty-nine Endocrine Disruptor Compounds-Pesticides in Rock, Soil, Water, Moss and Feces Samples from Antarctica in Gas Chromatography-Mass Spectrometry**

Buse Tuğba Zaman^a^, Gamze Dalgıç Bozyiğit^b^, Meltem Şaylan^a,c^ Elif Seda Koçoğlu^a^, Bedrihan Kartoğlu^a^, Efe Sinan Aydın^d^, Ayça Girgin^a,e^, Tülay Borahan^a,e^, Sude Oflu^a,e^, Yağmur Kılınç^e,f^, Gülhan Bakırdere^g^ Sezgin Bakırdere^a,h^
*^a^Yildiz Technical University, Faculty of Art and Science, Department of Chemistry, 34220, İstanbul, Türkiye*
*^b^Yildiz Technical University, Faculty of Civil Engineering, Department of Environmental Engineering, 34220, İstanbul, Türkiye*

*^c^İstanbul Health and Technology University, Department of Pharmacy, Seyitnizam Street, No: 85, İstanbul, Türkiye*
*^d^Yildiz Technical University, Faculty of Chemistry and Metallurgy, Department of Chemical Engineering, 34220, İstanbul, Türkiye*
*^e^Neutec Pharmaceuticals, Yildiz Technical University Technopark, 34220, İstanbul, Türkiye*
*^f^Zonguldak Bülent Ecevit University, Institute of Science, Department of Environmental Engineering, 67100, Zonguldak, Türkiye*

*^g^Yildiz Technical University, Faculty of Education, Department of Science Education, 34220, İstanbul, Türkiye*
*^h^Turkish Academy of Sciences (TÜBA), Vedat Dalokay Street, No. 112, Çankaya, 06670, Ankara, Türkiye*

**Supplemantary Informatiın**

**Tables**

**Table S1.** List of selected EDCs with specific characteristics and main quantifier ions used in this study.

| Number | Analyte | Brand | Analyte Type | Molecular weight, g/mol | The 5 major fragment ions with the highest peak area | Retention time, min |
| --- | --- | --- | --- | --- | --- | --- |
| 1 | alfa-benzenehexachloride (alfa-BHC) | Dr. Ehrenstorfer | Pesticide | 290.83 | **183**, 181, 219, 217, 111 | 7.900 |
| 2 | beta- benzenehexachloride (beta-BHC) | Dr. Ehrenstorfer | Pesticide | 290.83 | **219**, 181, 109, 183,217 | 8.425 |
| 3 | Lindane (gama-BHC) | Dr. Ehrenstorfer | Pesticide | 290.83 | **181**, 183, 219, 217, 109 | 8.553 |
| 4 | Diazinon | Sigma Aldrich | EDC | 304.35 | **179**, 137, 152, 199, 304 | 8.758 |
| 5 | Delta- benzenehexachloride (delta-BHC) | Dr. Ehrenstorfer | Pesticide | 290.83 | **109**, 219, 183, 181, 217 | 9.046 |
| 6 | 1,3,4,6,7,8-Hekzahydro-4,6,6,7,8,8-hekzame  (Galaxolide) | Sigma Aldrich | EDC | 258.4 | **243**, 213, 258, 244, 171 | 9.435 |
| 6 | 4-n-Nonylphenol | Sigma Aldrich | EDC | 220.35 | **107**, 220, 108, 77, 221 | 9.503 |
| 7 | Parathion Methyl | Sigma Aldrich | EDC | 263.21 | **109**, 263, 125, 79, 63 | 9.892 |
| 8 | Heptachlor | Sigma Aldrich |  | 373.32 | 100, **272**, 274, 270, 65 | 10.096 |
| 9 | Malathion | Sigma Aldrich | EDC | 330.36 | 125, **173**, 93, 127, 29 | 10.763 |
| 10 | Aldrin | Sigma Aldrich | Pesticide | 364.91 | 66, **263**, 265, 261, 91 | 10.947 |
| 11 | Triflumizole | Sigma Aldrich | EDC | 345.75 | 73, **278**, 206, 179, 287 | 12.504 |
| 12 | Paclobutrazol | Sigma Aldrich | EDC | 293.79 | **236**, 125, 82, 238, 57 | 12.847 |
| 13 | Triclosan methyl | Sigma Aldrich | Pesticide | 303.57 | **290,** 288, 218, 63, 51 | 12.936 |
| 14 | cis-chlordane | Sigma Aldrich | Pesticide | 409.78 | 373, **375**, 377, 371, 237 | 13.076 |
| 15 | Bisphenol A | Sigma Aldrich | EDC | 228.29 | **213**, 228, 119, 214, 91 | 13.590 |
| 16 | 4, 4-dicholorodiphenyl dichloroethylene (4,4-DDE) | Dr. Ehrenstorfer | Pesticide | 318.03 | **246**, 318, 248, 316, 176 | 13.686 |
| 17 | Dieldrin | Sigma Aldrich | Pesticide | 380.91 | **79**, 81, 82, 77, 263 | 13.749 |
| 18 | Endrin | Dr. Ehrenstorfer | Pesticide | 380.91 | **81**, 79, 263, 67, 82 | 14.391 |
| 19 | α – Endosulfan | Sigma Aldrich | Pesticide | 406.93 | **241,** 239, 195, 237, 339 | 14.684 |
| 19 | β – Endosulfan | Sigma Aldrich | Pesticide | 406.93 | 195, **241,** 237, 239, 207 | 14.684 |
| 20 | 4,4-dichlorodiphenyl dichloroethane (4,4-DDD) | Dr. Ehrenstorfer | Pesticide | 320.04 | **235**, 237, 165, 236, 199 | 14.950 |
| 21 | Endosulfan sulphate | Dr. Ehrenstorfer | Pesticide | 422.92 | **387**, 272, 274, 389, 385 | 15.980 |
| 22 | 4, 4- dichlorodiphenyl trichloroethane (4,4-DDT) | Dr. Ehrenstorfer | Pesticide | 354.49 | **235**, 237, 165, 236, 199 | 16.075 |
| 23 | Carbamazepine | Sigma Aldrich | EDC | 236.27 | **193**, 192, 236, 194, 191 | 16.240 |
| 24 | Endrin ketone | Dr. Ehrenstorfer | Pesticide | 380.91 | **67**, 317, 315, 319, 139 | 17.441 |
| 25 | Metoxychlor | Dr. Ehrenstorfer | Pesticide | 345.65 | **227**, 228, 212, 196, 195 | 17.950 |
| 26 | Estrone | Sigma Aldrich | EDC | 270.37 | **270,** 146, 185, 172, 145 | 20.064 |
| 27 | ß-Estradiol | Sigma Aldrich | EDC | 272.38 | **272**, 160, 146, 172, 159 | 20.251 |
| 28 | 17α-Ethynylestradiol | Sigma Aldrich | EDC | 296.40 | **213**, 296, 160, 133, 159 | 21.294 |
| 29 | Diltiazem HCl | Sigma Aldrich | EDC | 450.98 | **58**, 71, 72, 121, 150 | 25.506 |

***** Dark colours represent major ions, where quantitative determinations are made.

**Table S2.** Analytical figures of merit of the GC-MS system

| **Analyte** | **LOD, ng/g** | **LOQ, ng/g** | ***R*^2^** | | **Linear Range, ng/g** | **%RSD** |
| --- | --- | --- | --- | --- | --- | --- |
| **alfa-BHC** | 24.4 | 81.2 | | 0.9993 | 109.7 – 7331 | 11.8 |
| **beta-BHC** | 23.6 | 78.8 | | 0.9994 | 103.3 – 9079 | 13.0 |
| **Lindane (gama-BHC)** | 14.4 | 48.1 | | 0.9995 | 52.4 – 9367 | 13.4 |
| **Diazinon** | 25.6 | 85.5 | | 0.9983 | 102.2 – 19109 | 9.9 |
| **delta-BHC** | 89.9 | 299.6 | | 0.9985 | 531.4 – 19951 | 8.1 |
| **1,3,4,6,7,8-Hekzahidro-4,6,6,7,8,8-hekzam** | 72.0 | 240.0 | | 0.9989 | 396.6 – 15014 | 5.4 |
| **4-n-Nonylphenol** | 31.2 | 104.0 | | 0.9995 | 199.0 – 18930 | 4.4 |
| **Parathion Methyl** | 75.9 | 252.9 | | 0.9989 | 507.4 – 19209 | 17.3 |
| **Heptachlor** | 24.2 | 80.6 | | 0.9989 | 104.2 – 7016 | 9.3 |
| **Malathion** | 41.5 | 138.4 | | 0.9989 | 201.5– 19168 | 12.9 |
| **Aldrin** | 36.9 | 123.1 | | 0.9987 | 201.8 – 6921 | 4.0 |
| **Triflumizole** | 38.8 | 129.5 | | 0.9991 | 200.2 – 19049 | 10.5 |
| **Paclobutrazol** | 44.4 | 148.0 | | 0.9991 | 204.8 – 9236 | 9.1 |
| **Triklosan metil** | 106.4 | 354.5 | | 0.9996 | 488.2 – 18482 | 7.1 |
| **cis-chlordane** | 41.2 | 137.4 | | 0.9985 | 201.7 – 6916 | 3.9 |
| **Bisphenol A** | 60.8 | 202.6 | | 1.00 | 201.9 – 19202 | 6.9 |
| **4,4-DDE** | 19.1 | 63.6 | | 0.9984 | 101.8 – 8948 | 6.9 |
| **Dieldrin** | 28.4 | 94.5 | | 0.9988 | 103.2 – 6949 | 10.5 |
| **Endrin** | 52.9 | 176.2 | | 0.9986 | 504.9 -18958 | 2.6 |
| **Endosulfan** | 123.8 | 412.7 | | 0.9995 | 404.2 – 13860 | 7.6 |
| **4,4-DDD** | 9.3 | 31.0 | | 0.9987 | 52.2 – 19703 | 10.4 |
| **Endosulfan sulfate** | 43.0 | 143.3 | | 0.9997 | 205.6 – 38716 | 11.7 |
| **4,4-DDT** | 91.4 | 304.8 | | 0.9989 | 524.0 – 19675 | 8.5 |
| **Carbamazepine** | 201.0 | 669.9 | | 0.9997 | 1012 – 19386 | 13.4 |
| **Endrin ketone** | 62.0 | 206.6 | | 0.9986 | 203.5 – 19206 | 6.9 |
| **Methoxychlor** | 3.6 | 12.0 | | 0.9992 | 52.3 – 9354 | 6.9 |
| **Estrone** | 285.2 | 950.8 | | 0.9993 | 1007 – 19307 | 13.0 |
| **ß-Estradiol** | 419.7 | 1398.9 | | 0.9984 | 2034 – 19458 | 11.7 |
| **17α-Ethinylestradiol** | 56.9 | 189.9 | | 0.9991 | 201.5 – 19168 | 14.2 |
| **Diltiazem Hydrochloride** | 24.3 | 80.9 | | 0.9995 | 200.8 – 19103 | 5.3 |

*Total analyte concentration (α-Endosulfan + β-Endosulfan).

**Table S3.** Optimum values determined for variable parameters of SADF-LPME-GC-MS combined method for EDCs

| **Parameter** | **Conditions** |
| --- | --- |
| Sample Solution | 8.0 mL |
| Buffer solution/volume | pH 9.0/0.50 mL |
| Binary extraction solution type/ratio(v/v) | DCE:DCM (1:1) |
| Spray Repetition | 2 times |
| Vortexing period | 15 s |

**Table S4.** Percent recovery results using matrix matching approach on seawater sample coded HS-D7 *(n=4)*

| **Analyte** | **Spiked concentration, ng/g** | **%Recovery ± SD** |
| --- | --- | --- |
| **alfa-BHC** | 114.8 | 93.6 ± 3.5 |
|  | 175.4 | 96.5 ± 1.9 |
| **beta-BHC** | 114.8 | 92.3 ± 2.7 |
|  | 175.4 | 93.8 ± 1.5 |
| **Lindane (gama-BHC)** | 54.3 | 123.8 ± 1.9 |
|  | 114.8 | 90.4 ± 2.1 |
|  | 175.4 | 92.1 ± 1.2 |
| **Diazinon** | 97.6 | 101.8 ± 4.6 |
|  | 202.3 | 97.4 ± 1.6 |
|  | 493.3 | 93.6 ± 4.9 |
|  | 54.3 | 97.2 ± 4.4 |
| **delta-BHC** | 114.8 | 87.5 ± 4.3 |
|  | 175.4 | 93.4 ± 3.7 |
| **1,3,4,6,7,8-Hexahydro-4,6,6,7,8,8-hekzame** | 87.5 | 94.9 ± 2.0 |
|  | 159 | 94.1 ± 3.6 |
|  | 387.6 | 82.0 ± 7.2 |
| **4-n-Nonylphenol** | 97.6 | 102.6 ± 7.0 |
|  | 202.3 | 105.5 ± 3.3 |
|  | 493.3 | 100.1 ± 7.6 |
| **Parathion Methyl *** | 112 | 113.4 ± 8.0 |
|  | 203.4 | 88.8 ± 10.8 |
| **Heptachlor** | 114.1 | 95.0 ± 1.9 |
|  | 190.8 | 92.9 ± 1.3 |
|  | 462.6 | 77.7 ± 4.2 |
| **Malathion** | 111.8 | 97.3 ± 2.8 |
|  | 202.9 | 98.9 ± 3.8 |
|  | 494.8 | 98.7 ± 3.4 |
| **Aldrin** | 53.2 | 110.9 ± 2.1 |
|  | 113.4 | 89.4 ± 1.2 |
|  | 189.4 | 78.1 ± 2.8 |
| **Triflumizole** | 51.8 | 107.7 ± 8.2 |
|  | 111.1 | 91.3 ± 5.8 |
|  | 201.7 | 89.9 ± 3.8 |
|  | 422.8 | 78.7 ± 0.4 |
| **Paclobutrazol** | 113.6 | 99.6 ± 0.6 |
|  | 205.7 | 98.7 ± 2.2 |
|  | 503 | 81.8 ± 3.3 |
| **cis-chlordane** | 113.8 | 110.9 ± 4.8 |
|  | 184.7 | 98.0 ± 1.9 |
|  | 446.9 | 76.8 ± 1.0 |
| **Bisphenol A** | 112 | 98.6 ± 2.1 |
|  | 203.3 | 97.8 ± 2.1 |
|  | 495.7 | 100.2 ± 2.3 |
| **4,4-DDE** | 114.8 | 92.0 ± 1.6 |
|  | 175.4 | 98.6 ± 1.1 |
| **Dieldrin** | 53.3 | 125.0 ± 7.2 |
|  | 113.6 | 98.7 ± 1.1 |
|  | 189.8 | 94.5 ± 2.3 |
| **Endrin** | 112 | 122.1 ± 0.9 |
|  | 203.3 | 96.8 ± 2.7 |
| **Endosulfan** | 53.3 | 105.3 ± 5.0 |
|  | 113.5 | 98.8 ± 0.7 |
|  | 189.5 | 94.5 ± 1.8 |
|  | 459.5 | 89.4 ± 2.5 |
| **4,4-DDD** | 114.8 | 91.8 ± 2.6 |
|  | 175.4 | 94.8 ± 2.0 |
| **Endosulfan sulphate** | 54.3 | 110.1 ± 2.7 |
|  | 114.8 | 84.0 ± 3.2 |
|  | 175.4 | 91.4 ± 1.4 |
| **4,4-DDT** | 114.8 | 88.3 ± 2.2 |
|  | 175.4 | 96.4 ± 2.8 |
| **Carbamazepine** | 113 | 98.0 ± 1.5 |
|  | 205.2 | 94.2 ± 1.3 |
|  | 500.5 | 88.7 ± 2.6 |
| **Endrin Ketone*** | 54.3 | 122.4 ± 6.0 |
|  | 114.8 | 105.2 ± 0.4 |
|  | 175.4 | 110.4 ± 3.4 |
| **Metoxychlor** | 112 | 97.6 ± 3.1 |
|  | 203.3 | 92.8 ± 2.5 |
| **Estrone** | 113.5 | 92.9 ± 3.1 |
|  | 206 | 99.7 ± 1.9 |
|  | 502.3 | 94.7 ± 0.8 |
| **ß-Estradiol** | 112.6 | 102.3 ± 3.4 |
|  | 204.4 | 103.6 ± 2.1 |
|  | 498.4 | 97.2 ± 0.7 |
| **17α-Ethinylestradiol** | 52.2 | 127.7 ± 2.2 |
|  | 111.8 | 80.3 ± 1.0 |
|  | 202.9 | 91.7 ± 1.4 |
| **Diltiazem Hydrochloride** | 112 | 93.1 ± 2.2 |
|  | 203.3 | 96.7 ± 3.2 |
|  | 495.7 | 89.6 ± 3.7 |

**Exact match matrix matching was employed.*

**Table S5.** Percent recovery results using matrix matching approach on soil sample coded A *(n=4)*

| **Analyte** | **Spiked concentration, ng/g** | | **%Recovery ± SD** |
| --- | --- | --- | --- |
| **alfa-BHC** | | 54.6 | 82.5 ± 1.4 |
|  |  | 118.2 | 95.6 ± 5.5 |
|  |  | 162.4 | 100.5 ± 13.6 |
|  |  | 455.3 | 91.9 ± 5.4 |
| **beta-BHC** | | 118.2 | 81.6 ± 6.9 |
|  |  | 162.4 | 103.4 ± 4.6 |
|  |  | 455.3 | 96.8 ± 2.8 |
| **Lindane (gama-BHC)** | | 54.6 | 82.9 ± 1.4 |
|  |  | 118.2 | 92.5 ± 5.5 |
|  |  | 162.4 | 97.0 ± 7.3 |
|  |  | 455.3 | 85.1 ± 2.9 |
| **Diazinon** | | 52.2 | 97.6 ± 7.1 |
|  |  | 114.7 | 119.0 ± 9.2 |
|  |  | 187.2 | 107.9 ± 12.6 |
|  |  | 531.5 | 95.0 ± 5.0 |
|  | | 54.6 | 81.4 ± 0.7 |
| **delta-BHC** | | 118.2 | 86.0 ± 1.8 |
|  | | 162.4 | 87.7 ± 10.4 |
|  | | 455.3 | 79.5 ± 1.1 |
| **1,3,4,6,7,8-Hexahydro-4,6,6,7,8,8-hekzame (Galaxolide)** | | 90.1 | 98.2 ± 1.9 |
|  |  | 147.1 | 91.2 ± 8.7 |
|  |  | 417.6 | 80.8 ± 1.7 |
| **4-n-Nonylphenol** | | 51.7 | 76.6 ± 4.3 |
|  |  | 113.7 | 109.1 ± 11.3 |
|  |  | 185.5 | 102.2 ± 14.5 |
|  |  | 526.5 | 83.7 ± 4.5 |
| **Parathion Methyl** | | 188.2 | 79.2 ± 2.3 |
|  |  | 534.3 | 85.8 ± 5.5 |
| **Heptachlor** | | 53.9 | 84.8 ± 1.8 |
|  |  | 117.5 | 103.1 ± 3.8 |
|  |  | 176.5 | 99.6 ± 4.0 |
|  |  | 498.4 | 76.4 ± 3.9 |
| **Malathion** | | 52.4 | 75.5 ± 4.1 |
|  |  | 115.1 | 106.6 ± 2.0 |
|  |  | 187.8 | 103.1 ± 4.9 |
|  |  | 533.1 | 97.5 ± 5.7 |
| **Aldrin** | | 53.5 | 110.1 ± 1.8 |
|  |  | 116.7 | 115.8 ± 3.2 |
|  |  | 175.2 | 120.1±1.4 |
|  |  | 494.7 | 82.4±4.4 |
| **Triflumizole** | | 52.1 | 102.2 ± 3.9 |
|  |  | 114.4 | 91.1 ± 3.8 |
|  |  | 188.6 | 89.4 ± 5.5 |
|  |  | 455.5 | 87.5 ± 4.0 |
| **Paclobutrazol** | | 117 | 97.5 ± 7.0 |
|  |  | 190.3 | 101.5 ± 5.7 |
|  |  | 541.9 | 80.3 ± 3.4 |
| **cis-chlordane** | | 53.9 | 88.7 ± 4.6 |
|  |  | 117.2 | 81.4 ± 0.6 |
|  |  | 170.9 | 74.4 ± 1.3 |
| **Bisphenol A** | | 115.3 | 83.0 ± 0.6 |
|  |  | 188.1 | 81.4 ± 10.1 |
|  |  | 534.1 | 76.7 ± 4.0 |
| **4,4-DDE** | | 54.6 | 115.3 ± 10.5 |
|  |  | 118.2 | 103.8 ± 2.0 |
|  |  | 162.4 | 115.7 ± 13.8 |
| **Dieldrin** | | 53.6 | 113.0 ± 5.1 |
|  |  | 117 | 109.7 ± 6.7 |
|  |  | 175.6 | 115.9 ± 5.5 |
| **Endrin** | | 115.3 | 126.9 ± 7.1 |
|  |  | 188.1 | 99.6 ± 0.9 |
|  |  | 534.1 | 75.9 ± 2.0 |
| **Endosulfan** | | 53.6 | 95.4 ± 4.6 |
|  |  | 116.8 | 88.2 ± 2.4 |
|  |  | 175.4 | 120.1 ± 5.7 |
|  |  | 495 | 118.0 ± 5.0 |
| **4,4-DDD** | | 118.2 | 103.5 ± 3.0 |
|  |  | 162.4 | 115.2 ± 16.8 |
| **Endosulfan sulphate** | | 54.6 | 88.4 ± 11.3 |
|  |  | 118.2 | 110.7 ± 15.5 |
|  |  | 162.4 | 113.7 ± 14.6 |
|  |  | 455.3 | 100.4 ± 4.4 |
| **4,4-DDT** | | 54.6 | 105.4 ± 5.8 |
|  |  | 118.2 | 107.9 ± 6.6 |
|  |  | 162.4 | 116.5 ± 11.1 |
|  |  | 455.3 | 102.9 ± 5.1 |
| **Carbamazepine** | | 116.4 | 83.4 ± 6.5 |
|  |  | 189.9 | 130.3 ± 9.3 |
|  |  | 539.2 | 116.7 ± 4.8 |
| **Endrin ketone** | | 54.6 | 92.1 ± 8.7 |
|  |  | 118.2 | 116.7 ± 8.9 |
|  |  | 162.4 | 123.4 ± 19.4 |
|  |  | 455.3 | 80.7 ± 4.3 |
| **Methoxychlor** | | 52.5 | 74.7 ± 9.2 |
|  |  | 115.3 | 111.1 ± 9.3 |
|  |  | 188.1 | 114.6 ± 2.2 |
| **Estrone** | | 116.8 | 77.6 ± 3.1 |
|  |  | 190.7 | 82.9 ± 13.3 |
|  |  | 541.2 | 85.8 ± 7.6 |
| **ß-Estradiol** | | 115.9 | 89.5 ± 3.0 |
|  |  | 189.2 | 88.5 ± 12.2 |
|  |  | 537 | 87.5 ± 6.9 |
| **17α-Ethinylestradiol** | | 52.4 | 78.5 ± 3.5 |
|  |  | 115.1 | 88.3 ± 3.9 |
|  |  | 187.8 | 80.7 ± 2.7 |
|  |  | 533.1 | 92.7 ± 5.1 |
| **Diltiazem Hydrochloride** | | 115.3 | 79.4 ± 2.3 |
|  |  | 188.1 | 92.1 ± 2.4 |
|  |  | 534.1 | 73.7 ± 5.2 |

**Exact match matrix matching was employed.*

**Table S6.** Percent recovery results using matrix matching approach on soil sample coded B *(n=4)*

| **Analyte** | **Spiked concentration, ng/g** | **%Recovery ± SD** |
| --- | --- | --- |
| **alfa-BHC** | 62.7 | 100.5 ± 7.5 |
|  | 104.3 | 113.3 ± 12.5 |
|  | 201 | 83.9 ± 6.3 |
| **beta-BHC** | 104.3 | 118.3 ± 25.1 |
|  | 201 | 114.8 ± 12.7 |
|  | 419.2 | 87.6 ± 3.6 |
| **Lindane (gama-BHC)** | 62.7 | 97.9 ± 1.2 |
|  | 104.3 | 118.1 ± 10.9 |
|  | 201 | 79.6 ± 3.4 |
| **Diazinon** | 60 | 83.2 ± 3.4 |
|  | 101.2 | 105.5 ± 7.8 |
|  | 231.7 | 93.1 ± 1.9 |
|  | 489.3 | 105.0 ± 3.4 |
| **delta-BHC** | 62.7 | 100.3 ± 16.5 |
|  | 104.3 | 110.9 ± 4.8 |
|  | 419.2 | 123.6 ± 5.3 |
| **1,3,4,6,7,8-Hexahydro-4,6,6,7,8,8-hekzame (Galaxolide)** | 47.1 | 74.1 ± 3.8 |
|  | 384.5 | 122.3 ± 3.2 |
| **4-n-Nonylphenol** | 59.4 | 82.0 ± 4.7 |
|  | 229.6 | 129.1 ± 2.0 |
|  | 484.7 | 113.7 ± 5.1 |
| **Parathion Methyl *** | 101.8 | 102.6 ± 2.8 |
|  | 233 | 123.4 ± 0.2 |
| **Heptachlor** | 61.9 | 103.8 ± 3.9 |
|  | 103.7 | 100.0 ± 5.1 |
|  | 218.5 | 105.3 ± 1.0 |
| **Malathion** | 60.2 | 119.2 ± 7.4 |
|  | 101.6 | 118.9 ± 10.1 |
|  | 232.5 | 87.8 ± 1.2 |
|  | 490.8 | 100.9 ± 4.2 |
| **Aldrin** | 61.5 | 78.9 ± 3.7 |
|  | 103 | 93.0 ± 3.1 |
|  | 216.9 | 119.5 ± 2.7 |
| **Triflumizole** | 59.8 | 89.0 ± 5.4 |
|  | 100.9 | 118.0 ± 9.8 |
|  | 231 | 89.2 ± 0.9 |
|  | 419.4 | 114.1 ± 5.6 |
| **Paclobutrazol** | 61.2 | 84.1 ± 3.1 |
|  | 103.2 | 111.0 ± 1.1 |
|  | 235.6 | 105.1 ± 4.1 |
| **cis-chlordane** | 19.7 | 99.2 ± 4.0 |
|  | 61.9 | 97.2 ± 2.2 |
| **Bisphenol A** | 62.7 | 107.2 ± 3.9 |
|  | 104.3 | 129.8 ± 2.0 |
|  | 201 | 127.4 ± 4.1 |
| **4,4-DDE** | 62.7 | 86.6 ± 2.2 |
|  | 104.3 | 105.6 ± 9.5 |
|  | 201 | 101.2 ± 25.4 |
| **Dieldrin** | 61.6 | 77.5 ± 0.7 |
|  | 103.2 | 89.6 ± 4.2 |
|  | 217.4 | 87.9 ± 0.7 |
| **Endrin** | 201 | 111.2 ± 3.7 |
|  | 419.2 | 130.8 ± 2.4 |
| **Endosulfan** | 61.5 | 119.8 ± 3.8 |
|  | 103.1 | 110.5 ± 8.6 |
|  | 217.1 | 90.0 ± 4.3 |
|  | 455.7 | 84.7 ± 4.6 |
| **4,4-DDD** | 62.7 | 105.5 ± 4.0 |
|  | 104.3 | 113.1± 13.3 |
|  | 201 | 87.5 ± 3.5 |
| **Endosulfan sulphate** | 62.7 | 96.0 ± 1.1 |
|  | 104.3 | 96.2 ± 12.5 |
|  | 201 | 111.0 ± 2.2 |
| **4,4-DDT** | 62.7 | 107.9 ± 1.0 |
|  | 104.3 | 104.2 ± 5.0 |
|  | 201 | 80.8 ± 11.0 |
| **Carbamazepine** | 102.7 | 115.0 ± 2.7 |
|  | 235.1 | 81.6 ± 1.0 |
| **Endrin Ketone** | 104.3 | 96.8 ± 17.1 |
|  | 201 | 79.6 ± 14.0 |
| **Methoxychlor** | 62.7 | 109.9 ± 1.8 |
|  | 104.3 | 116.1 ± 2.5 |
|  | 201 | 92.1 ± 3.2 |
| **Estrone** | 61.1 | 92.3 ± 5.7 |
|  | 498.3 | 115.1 ± 2.7 |
| **ß-Estradiol** | 60.6 | 91.9 ± 5.9 |
|  | 102.3 | 117.1 ± 8.2 |
|  | 494.4 | 113.5 ± 2.6 |
| **17α-Ethinylestradiol** | 101.6 | 130.5 ± 4.5 |
|  | 232.5 | 101.2 ± 8.4 |
|  | 490.8 | 110.3 ± 5.4 |
| **Diltiazem Hydrochloride** | 62.7 | 87.9 ± 0.5 |
|  | 201 | 127.4 ± 10.0 |

*Exact match matrix matching was employed.

**Table S7.** Percent recovery results using matrix matching approach on moss sample coded A *(n=4)*

| **Analyte** | **Spiked concentration, ng/g** | **%Recovery ± SD** |
| --- | --- | --- |
| **alfa-BHC** | 52.4 | 112.8 ± 1.2 |
|  | 104.7 | 120.8 ± 22.3 |
| **beta-BHC** | 52.4 | 108.6 ± 3.5 |
|  | 104.7 | 101.1 ± 3.9 |
|  | 173.7 | 113.1 ± 6.1 |
| **Lindane (gama-BHC)** | 52.4 | 94.4 ± 1.5 |
|  | 104.7 | 91.8 ± 16.2 |
|  | 173.7 | 105.7 ± 3.2 |
| **Diazinon** | 50.1 | 92.0 ± 1.6 |
|  | 101.7 | 107.2 ± 4.3 |
|  | 200.2 | 99.8 ± 4.7 |
|  | 483.9 | 103.3 ± 5.1 |
| **delta-BHC** | 52.4 | 108.4 ± 7.4 |
|  | 104.7 | 104.9 ± 6.2 |
|  | 173.7 | 119.5 ± 7.0 |
|  | 414.6 | 109.0 ± 1.1 |
| **1,3,4,6,7,8-Hexahydro-4,6,6,7,8,8-hekzame (Galaxolide)** | 157.3 | 91.2 ± 0.8 |
|  | 380.2 | 84.4 ± 5.5 |
| **4-n-Nonylphenol *** | 100.7 | 84.3 ± 5.9 |
|  | 198.4 | 104.3 ± 19.1 |
| **Parathion Methyl *** | 102.2 | 98.6 ± 7.9 |
|  | 201.3 | 106.3 ± 5.8 |
| **Heptachlor *** | 104.1 | 92.2 ± 3.3 |
|  | 188.8 | 95.2 ± 6.2 |
| **Malathion** | 102 | 126.9 ± 17.4 |
|  | 200.9 | 115.7 ± 7.4 |
|  | 485.4 | 99.1 ± 3.0 |
| **Aldrin** | 51.4 | 120.5 ± 2.3 |
|  | 103.4 | 102.9 ± 2.8 |
|  | 187.4 | 105.1 ± 7.5 |
|  | 450.4 | 92.1 ± 0.5 |
| **Triflumizole** | 50 | 100.3 ± 5.4 |
|  | 101.3 | 99.5 ± 4.9 |
|  | 199.6 | 86.6 ± 11.7 |
|  | 414.8 | 85.8 ± 3.8 |
| **Paclobutrazol** | 51.1 | 92.6 ± 3.1 |
|  | 103.6 | 104.8 ± 5.0 |
|  | 203.6 | 114.8 ± 1.8 |
|  | 493.4 | 110.8 ± 2.8 |
| **cis-chlordane** | 103.9 | 105.5 ± 6.3 |
|  | 182.8 | 96.6 ± 2.7 |
| **Bisphenol A** | 50.4 | 114.9 ± 1.9 |
|  | 102.2 | 111.8 ± 3.7 |
|  | 201.2 | 105.3 ± 1.8 |
|  | 486.3 | 91.7 ± 1.4 |
| **4,4-DDE*** | 52.4 | 122.0 ± 14.2 |
|  | 104.7 | 110.5 ± 4.9 |
| **Dieldrin*** | 103.7 | 116.2 ± 8.0 |
|  | 187.8 | 107.6 ± 10.0 |
| **Endrin** | 201.2 | 106.5 ± 2.8 |
|  | 486.3 | 86.0 ± 7.0 |
| **Endosulfan** | 187.6 | 114.9 ± 4.4 |
|  | 450.7 | 94.3 ± 4.9 |
| **4,4-DDD** | 104.7 | 82.4 ± 9.9 |
|  | 173.7 | 97.5 ± 6.1 |
|  | 414.6 | 87.9 ± 1.0 |
| **Endosulfan sulphate** | 52.4 | 116.1 ± 8.8 |
|  | 104.7 | 113.6 ± 9.0 |
|  | 173.7 | 80.6 ± 3.4 |
| **4,4-DDT** | 104.7 | 122.2 ± 12.2 |
|  | 173.7 | 105.3 ± 2.6 |
|  | 414.6 | 77.6 ± 5.5 |
| **Carbamazepine** | 50.8 | 100.9 ± 6.2 |
|  | 103.1 | 110.4 ± 4.9 |
|  | 203.2 | 102.3 ± 7.9 |
|  | 491 | 91.6 ± 1.9 |
| **Endrin Ketone*** | 173.7 | 95.5 ± 4.7 |
| **Methoxychlor** | 50.4 | 96.6 ± 4.8 |
|  | 102.2 | 120.2 ± 7.2 |
|  | 201.2 | 89.5 ± 0.5 |
|  | 486.3 | 87.4 ± 4.7 |
| **Estrone** | 51 | 88.3 ± 2.6 |
|  | 103.5 | 99.4 ± 7.2 |
|  | 203.9 | 106.7 ± 5.6 |
|  | 492.8 | 100.8 ± 4.3 |
| **ß-Estradiol** | 102.7 | 93.0 ± 7.5 |
|  | 202.3 | 104.5 ± 5.9 |
|  | 489 | 101.5 ± 4.0 |
| **17α-Ethinylestradiol** | 50.3 | 117.9 ± 9.3 |
|  | 102 | 122.5 ± 1.0 |
|  | 200.9 | 111.3 ± 1.8 |
|  | 485.5 | 102.3 ± 2.0 |
| **Diltiazem Hydrochloride** | 201.2 | 116.0 ± 7.8 |
|  | 486.3 | 108.5 ± 2.9 |

* Exact match matrix matching was employed.

**Table S8.** Percent recovery results using matrix matching approach on moss sample coded B *(n=4)*

| **Analyte** | **Spiked concentration, ng/g** | **%Recovery ± SD** |  |
| --- | --- | --- | --- |
| **alfa-BHC** | 51.6 | 101.6 ± 2.8 |  |
|  | 102.7 | 80.6 ± 3.5 |  |
| **beta-BHC** | 51.6 | 101.5 ± 0.7 |  |
|  | 102.7 | 84.7 ± 3.5 |  |
|  | 196.5 | 91.8 ± 3.9 |  |
| **Lindane (gama-BHC)** | 51.6 | 107.2 ± 0.1 |  |
|  | 102.7 | 91.9 ± 1.1 |  |
|  | 196.5 | 98.7 ± 6.1 |  |
| **Diazinon** | 49.4 | 98.1 ± 8.8 |  |
|  | 99.7 | 108.5 ± 3.9 |  |
|  | 226.6 | 93.3 ± 3.7 |  |
|  | 473.1 | 97.7 ± 0.6 |  |
| **delta-BHC** | 51.6 | 86.5 ± 1.9 |  |
|  | 102.7 | 82.1 ± 1.8 |  |
|  | 196.5 | 95.3 ± 1.8 |  |
|  | 405.3 | 90.0 ± 2.5 |  |
| **1,3,4,6,7,8-Hexahydro-4,6,6,7,8,8-hekzame (Galaxolide)** | 38.8 | 94.9 ± 1.8 |  |
|  | 78.3 | 79.2 ± 2.2 |  |
|  | 178 | 108.6 ± 1.8 |  |
|  | 371.7 | 121.7 ± 0.9 |  |
| **4-n-Nonylphenol *** | 98.7 | 99.4 ± 8.4 |  |
|  | 224.5 | 91.2 ± 1.8 |  |
| **Parathion Methyl *** | 102.2 | 80.0 ± 4.9 |  |
|  | 227.8 | 112.9 ± 1.4 |  |
| **Heptachlor *** | 102.1 | 83.1 ± 4.4 |  |
|  | 213.7 | 102.3 ± 3.1 |  |
| **Malathion** | 100 | 81.4 ± 3.5 |  |
|  | 227.3 | 91.9 ± 3.9 |  |
|  | 474.5 | 99.5 ± 1.2 |  |
| **Aldrin** | 50.6 | 99.1 ± 2.4 |  |
|  | 101.4 | 81.6 ± 1.4 |  |
|  | 212.1 | 92.8 ± 5.7 |  |
|  | 440.3 | 107.9 ± 3.0 |  |
| **Triflumizole** | 99.3 | 105.6 ± 1.0 |  |
|  | 225.9 | 102.3 ± 8.4 |  |
|  | 405.5 | 120.4 ± 5.4 |  |
| **Paclobutrazol** | 50.4 | 96.6 ± 1.7 |  |
|  | 101.6 | 99.3 ± 1.8 |  |
|  | 230.4 | 88.5 ± 1.8 |  |
|  | 482.3 | 90.2 ± 0.7 |  |
| **cis-chlordane** | 50.9 | 75.9 ± 7.2 |  |
|  | 206.9 | 100.7 ± 4.2 |  |
| **Bisphenol A** | | 49.6 | 91.9 ± 9.9 |
|  |  | 100.1 | 99.4 ± 5.7 |
|  |  | 227.7 | 99.2 ± 3.2 |
|  |  | 475.4 | 108.2 ± 5.8 |
| **4,4-DDE*** | | 51.6 | 114.3 ± 4.4 |
|  |  | 102.7 | 94.1 ± 2.5 |
| **Dieldrin*** | | 101.6 | 92.0 ± 6.9 |
|  |  | 212.6 | 93.7 ± 11.1 |
| **Endrin** | | 227.7 | 92.6 ± 5.0 |
|  |  | 475.4 | 120.1 ± 6.6 |
| **Endosulfan** | | 212.3 | 83.1 ± 5.3 |
|  |  | 440.6 | 107.6 ± 1.9 |
| **4,4-DDD** | | 51.6 | 92.9 ± 4.0 |
|  |  | 196.5 | 114.6 ± 14.4 |
|  |  | 405.3 | 108.1 ± 4.6 |
| **Endosulfan sulphate** | | 102.7 | 106.4 ± 8.0 |
|  |  | 196.5 | 128.2 ± 5.7 |
| **4,4-DDT** | | 196.5 | 113.3 ± 12.0 |
|  |  | 405.3 | 132.4 ± 15.9 |
| **Carbamazepine** | | 50.1 | 79.3 ± 0.8 |
|  |  | 101.1 | 99.2 ± 0.5 |
|  |  | 229.9 | 97.5 ± 2.7 |
|  |  | 479.9 | 109.6 ± 3.7 |
| **Endrin Ketone*** | | 102.7 | 88.6 ± 0.4 |
|  |  | 196.5 | 97.7 ± 4.8 |
| **Methoxychlor** | | 49.6 | 78.4 ± 7.3 |
|  |  | 100.1 | 99.6 ± 2.8 |
|  |  | 227.7 | 121.8 ± 9.3 |
|  |  | 475.4 | 112.3 ± 6.7 |
| **Estrone** | | 50.3 | 91.3 ± 6.4 |
|  |  | 101.5 | 123.5 ± 28.6 |
|  |  | 230.7 | 90.4 ± 6.2 |
|  |  | 481.7 | 99.5 ± 3.6 |
| **ß-Estradiol** | | 49.9 | 91.9 ± 9.0 |
|  |  | 229 | 89.9 ± 6.3 |
|  |  | 478 | 99.1 ± 3.5 |
| **17α-Ethinylestradiol** | | 100 | 89.5 ± 0.5 |
|  |  | 227.3 | 89.4 ± 2.9 |
|  |  | 474.5 | 97.5 ± 0.7 |
| **Diltiazem Hydrochloride** | | 100.1 | 88.8 ± 7.6 |
|  |  | 227.7 | 93.8 ± 2.7 |
|  |  | 475.4 | 89.1± 2.5 |

* Exact match matrix matching was employed.

**Figures**


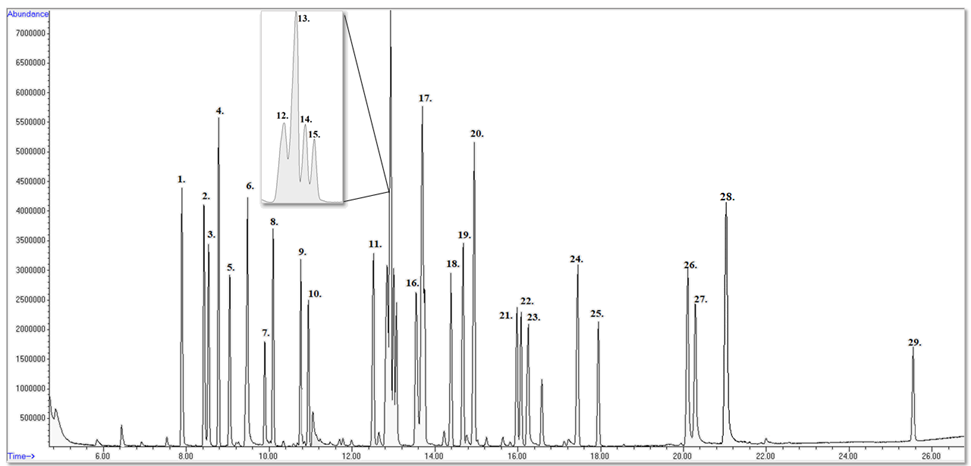


**Fig.S1** Total ion chromatogram of 50 mg/kg mixed standard EDCs solution obtained GC-MS.


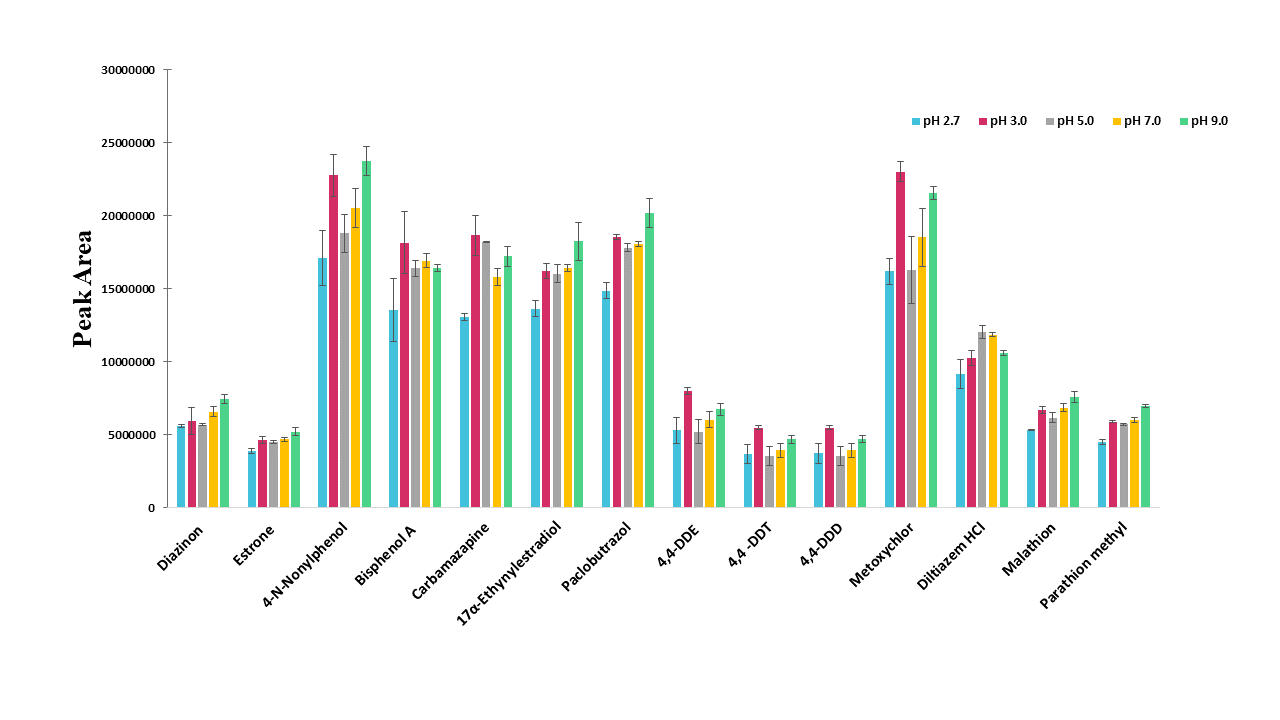

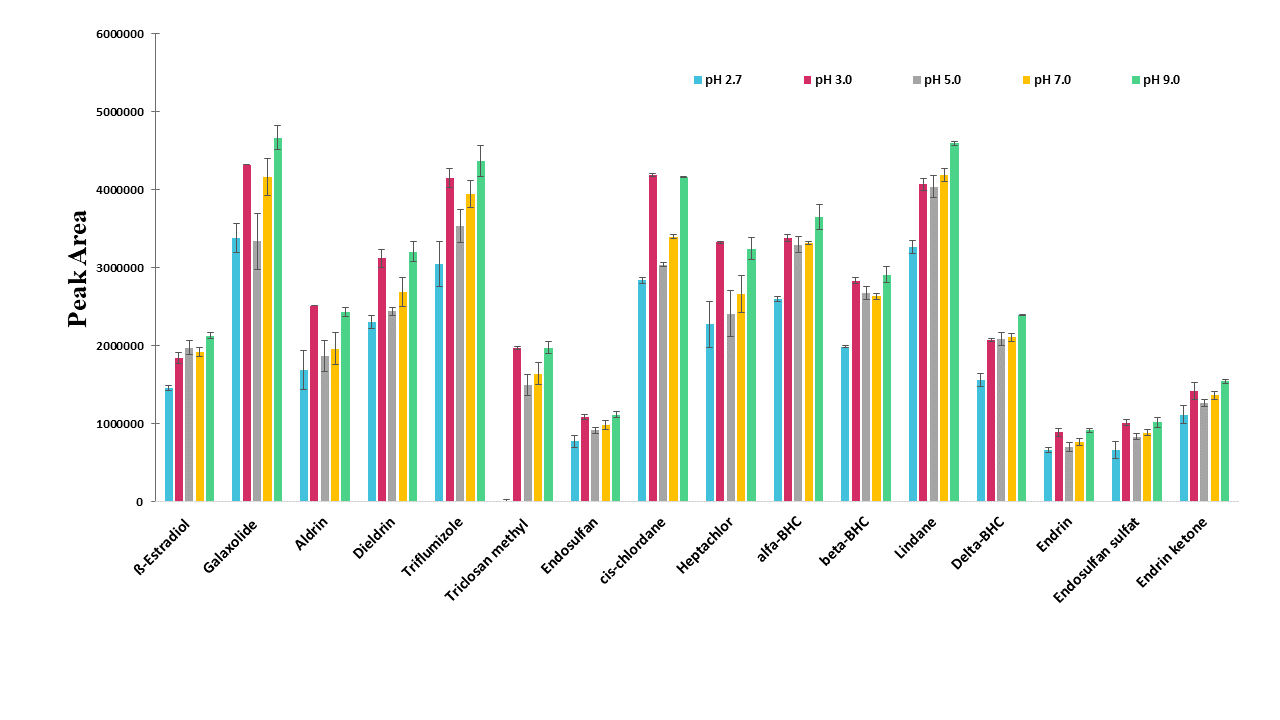


**Fig.S2.** Effect of the pH of the sample solution for the determination of the selected EDCs (Conditions: 5.0 mL of sample solution, 1.0 mL buffer solution, 2 spray repetition of CHL, and 15 s vortex, n=4).


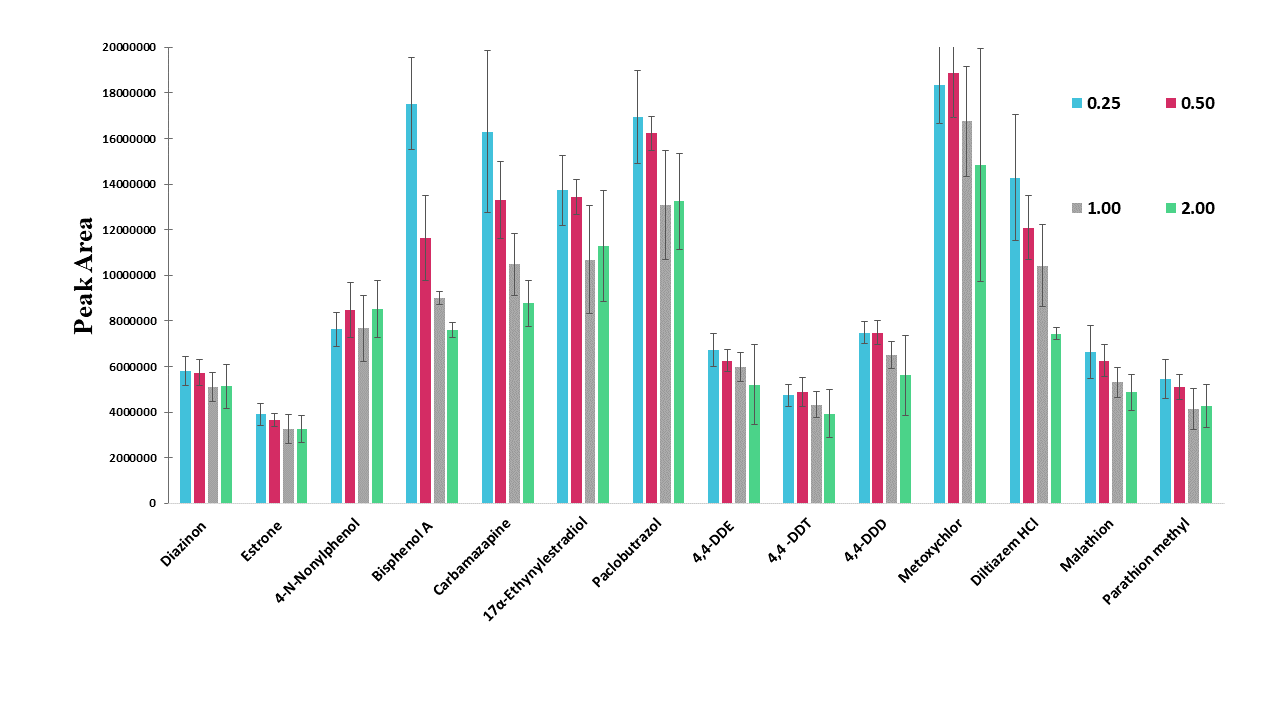


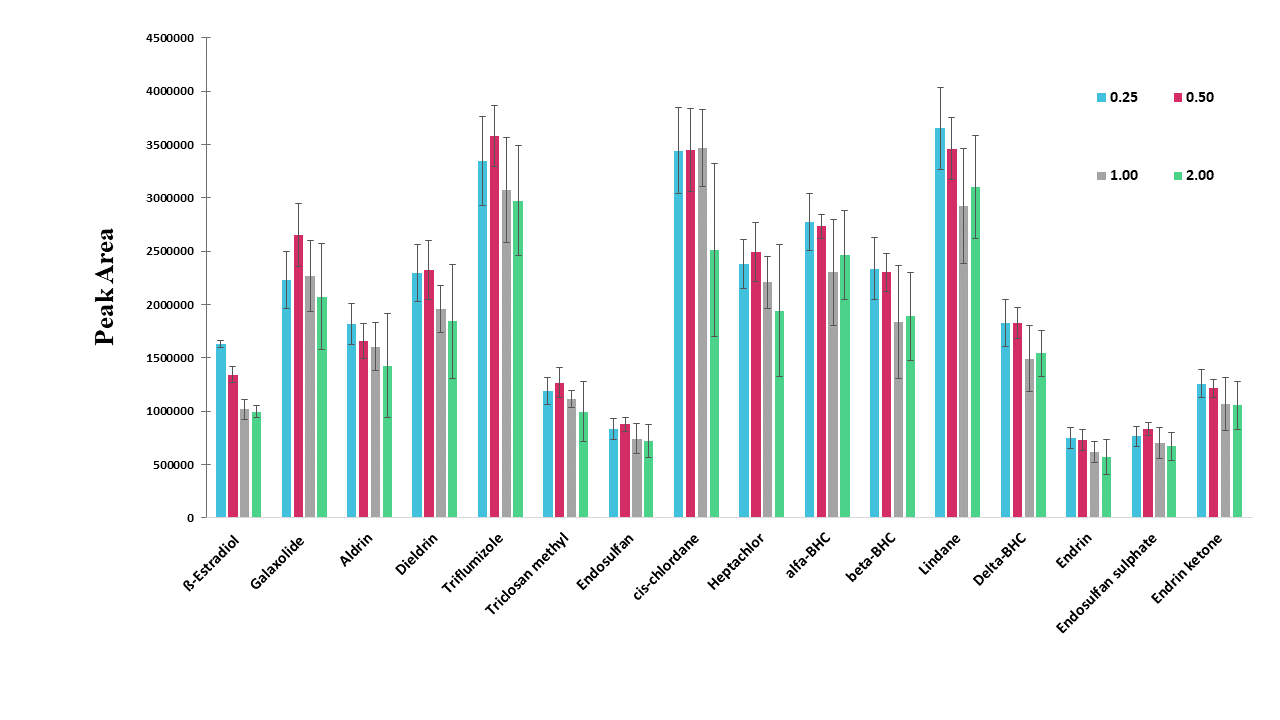


**Fig.S3.** Effect of the volume of the buffer solution for the determination of the selected EDCs (Conditions: 5.0 mL of sample solution, borate-HCl buffer solution at pH 9.0, 2 spray repetition of CHL, and 15 s vortex, n=4).


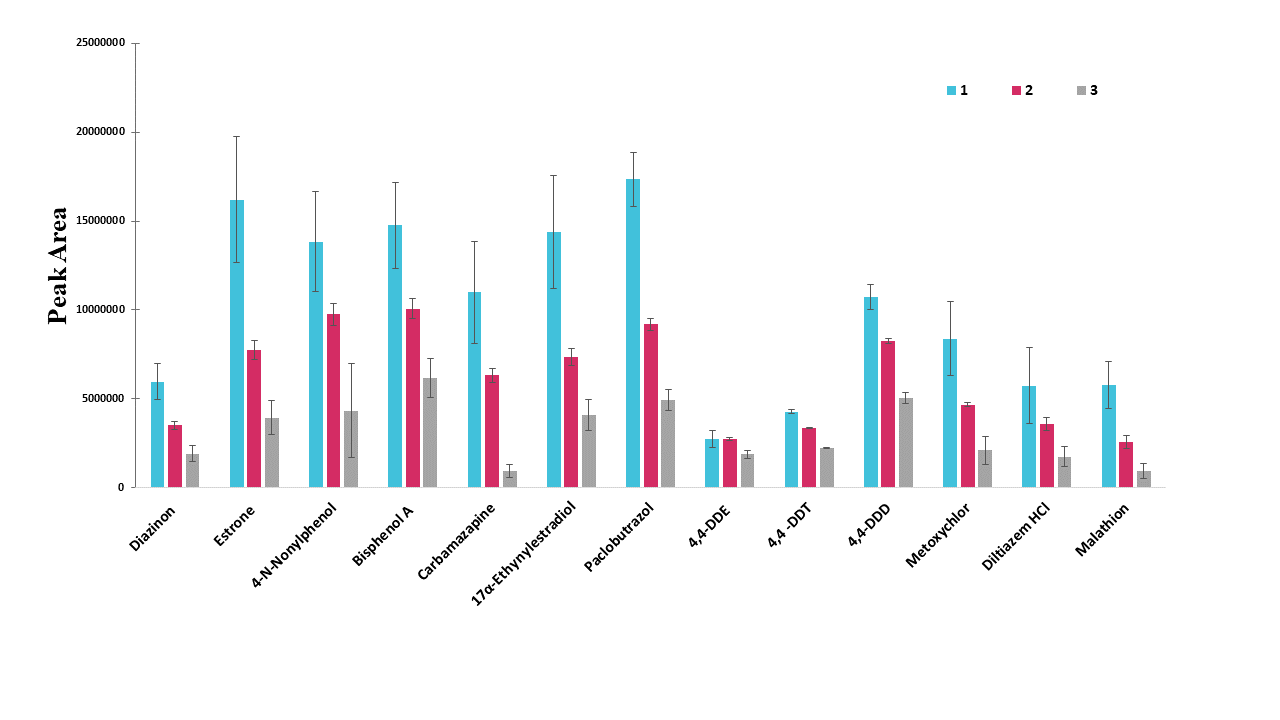


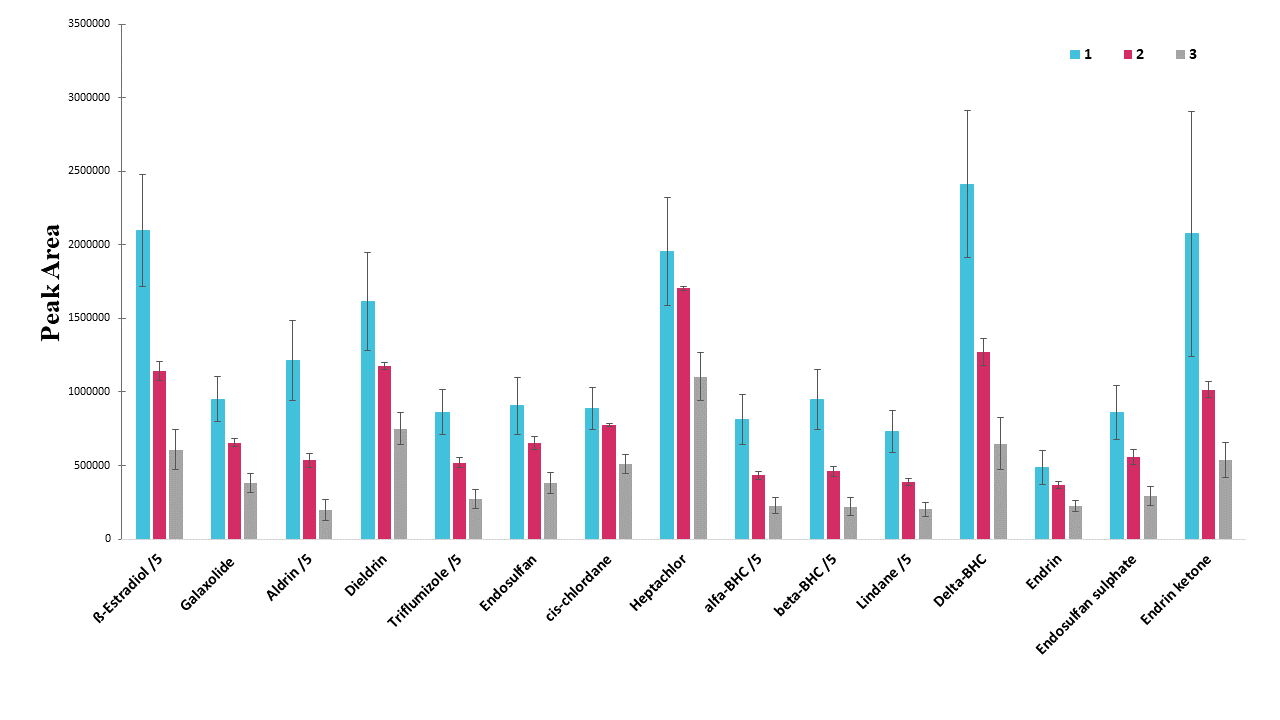


**Fig.S4.** Effect of the spray number (extraction solvent volume) for the determination of the selected EDCs (Conditions: 5.0 mL of sample solution, 0.50 mL borate-HCl buffer solution at pH 9.0, DCE-DCM solvent mixture (1:1, v/v) as extraction solvent, and 15 s vortex, n=4).


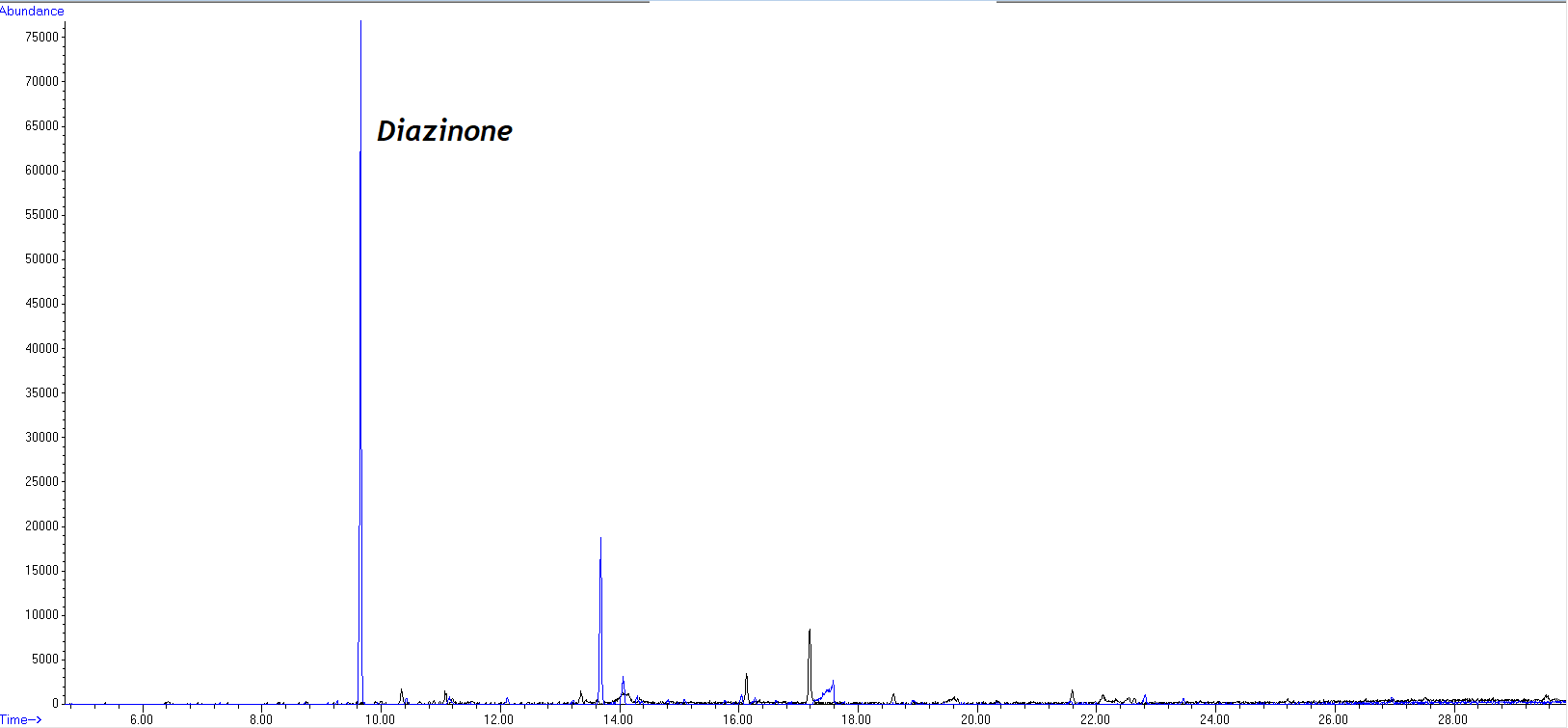


**Fig. S5.** Selected ion chromatograms obtained using the diazinon analyte of a seaweed sample collected from Horseshoe Island and a 5000 ng/g mixed standard solution of the same sample (m/z: 179)
